# Exploring the Chemical Space of Urease Inhibitors to Extract Meaningful Trends and Drivers of Activity

**DOI:** 10.1021/acs.jcim.2c00150

**Published:** 2022-06-06

**Authors:** Natália Aniceto, Vasco D. B. Bonifácio, Rita C. Guedes, Nuno Martinho

**Affiliations:** † Research Institute for Medicines (iMed.ULisboa), Faculty of Pharmacy, Universidade de Lisboa, 1649-003 Lisbon, Portugal; ‡ Department of Pharmaceutical Sciences and Medicines, Faculty of Pharmacy, Universidade de Lisboa, 1649-003 Lisbon, Portugal; § iBB—Institute for Bioengineering and Biosciences, Instituto Superior Técnico, Universidade de Lisboa, Av. Rovisco Pais, 1049-001 Lisboa, Portugal; ∥ Associate Laboratory i4HB—Institute for Health and Bioeconomy at Instituto Superior Técnico, Universidade de Lisboa, Av. Rovisco Pais, 1049-001 Lisboa, Portugal; ⊥ Department of Bioengineering, Instituto Superior Técnico, Universidade de Lisboa, Av. Rovisco Pais, 1049-001 Lisboa, Portugal

## Abstract

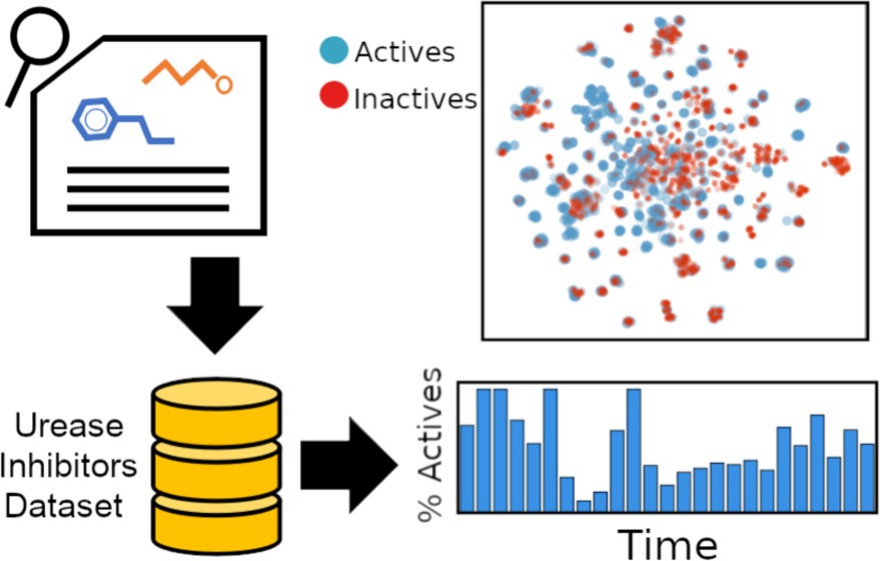

Blocking the catalytic activity of urease has been shown to have a key role in different diseases as well as in different agricultural applications. A vast array of molecules have been tested against ureases of different species, but the clinical translation of these compounds has been limited due to challenges of potency, chemical and metabolic stability as well as promiscuity against other proteins. The design and development of new compounds greatly benefit from insights from previously tested compounds; however, no large-scale studies surveying the urease inhibitors’ chemical space exist that can provide an overview of developed compounds to data. Therefore, given the increasing interest in developing new compounds for this target, we carried out a comprehensive analysis of the activity landscape published so far. To do so, we assembled and curated a data set of compounds tested against urease. To the best of our knowledge, this is the largest data set of urease inhibitors to date, composed of 3200 compounds of diverse structures. We characterized the data set in terms of chemical space coverage, molecular scaffolds, distribution with respect to physicochemical properties, as well as temporal trends of drug development. Through these analyses, we highlighted different substructures and functional groups responsible for distinct activity and inactivity against ureases. Furthermore, activity cliffs were assessed, and the chemical space of urease inhibitors was compared to DrugBank. Finally, we extracted meaningful patterns associated with activity using a decision tree algorithm. Overall, this study provides a critical overview of urease inhibitor research carried out in the last few decades and enabled finding underlying SAR patterns such as under-reported chemical functional groups that contribute to the overall activity. With this work, we propose different rules and practical implications that can guide the design or selection of novel compounds to be screened as well as lead optimization.

## Introduction

Urease, a metalloenzyme containing an active site with two nickel ions, is responsible for the catalytic hydrolysis of urea into ammonia and carbamate. It is found and conserved in many organisms among plants, bacteria, and fungi and plays an important role in nitrogen metabolism.^1^ Urease inhibition is therefore highly desirable for different applications in fields such as agriculture to control nitrogen loss^2,3^ and medicine, to treat bacterial infections.^4,5^ For the latter, urease acts as an important virulent factor, particularly in the pathogenesis of gastric infection by*Helicobacter pylori*, as well as in infectious urolithiasis (development of urinary stones) and catheter blockage by*Proteus mirabilis*.^6,7^ As of 2020, according to the CDC, two-thirds of the world population is infected with*H. pylori*.^8^ This microorganism is one of the main causes of gastritis and stomach ulcers, and a major risk factor for the development of gastric cancer. The eradication of *H. pylori* using antibiotics has been shown to improve all of those outcomes.^9^ Unfortunately, antimicrobial resistance by this bacterium has been rising over the last decade and in 2017 the WHO declared the development of antibiotics against clarithromycin-resistant *H. pylori* a high-priority issue.^9^ Regarding *P. mirabilis*, this is one of the main agents in catheter-associated urinary tract infections, which can lead to septicemia and endotoxic shock. Like *H. pylori*, *P. mirabilis* is also associated with antimicrobial resistance.^7^ As a result, the discovery of new antimicrobial molecules against this pathogen is of high priority, particularly those targeting urease, since this protein plays an essential role in the survival of these and other bacteria.

Over the last few decades, many compounds have been tested against ureases of different species.^4,10,11^ The inhibition mechanisms have been found to be mostly *via* direct binding to the active site bearing the nickel ions or by blocking the mobile flap as observed for covalent inhibitors that bind to a specific cysteine present in a mobile “flap” at the entry of the active site.^4,11^ Despite detailed knowledge of the crystal structure of the active site of many ureases, even in the presence of known inhibitors, the clinical translation of urease inhibitors has been limited. Even though highly potent inhibitors have been found, the limitations observed *in vivo* are mostly due to chemical and metabolic instability as well as toxicity of the compounds. In fact, acetohydroxamic acid, which has been clinically approved for the treatment of urinary infections, has significant side effects. Finally, compounds that would need to inhibit *H. pylori* urease need to survive the harsh hydrolytic conditions of the stomach.^12^ Consequently, there is still a need for the continued development of novel urease inhibitors for medical application.

Perhaps due to the shortcomings of known urease inhibitors, a wealth of diverse chemical scaffolds with inhibitory activity has been published targeting the different ureases.^3,4,10,11,13^ Since the active site of ureases is well conserved among different species,^14,15^ it is highly desirable to take into account information from different species to serve as a starting point for the development of novel inhibitors that may have appropriate pharmaceutical use. However, the exhaustive analysis of all publicly available urease inhibitors has yet to be carried out. Mostly, a small number of studies have focused on specific structure-activity relationships of small subsets with a low diversity of compounds. Such a large-scale analysis of the inhibitors accumulated in the public domain over the last few decades is key to drawing insights into the structural patterns that drive urease inhibition and to enable rational, evidence-based drug design and discovery.

To meet this need, in the current work we carried out an exhaustive retrieval and curation of activity data on urease inhibitors comprising 3200 small-molecule urease inhibitors against different species, and used it to perform a comprehensive analysis. We focused on a data-driven strategy whereby we identified important structural and physicochemical determinants that can guide future drug discovery of new lead compounds and tune their pharmacological properties, saving time and experimental efforts.

## Methods

### Collection and Curation of Urease Inhibitors and Annotation with Molecular Descriptors

A data set comprising 4122 raw activities against urease was assembled from data retrieved from publicly available literature (up to April 2021), patents, and ChEMBL 28.^16^ In this data set, we collated inhibition data for ureases from different species, although Jack bean and *H. pylori* make up ∼80% of the data. This was done to maximize the analysis of investigated urease inhibitors so far and because actives can potentially be transferable between different ureases, owing to their high binding site similarity. We also included a small portion of assays where authors did not specify which urease they used (11% of the full data). Table 1 shows a breakdown of all species and their contributions.

**Table 1:** Species for which Urease Inhibition Data Was Gathered and Their Corresponding Compound Contribution

species	*N* unique compounds	% compounds
*Canavalia ensiformis* (Jack bean)	2187	68.34
unknown	376	11.75
*H. pylori*	317	9.91
*Sporosarcina pasteurii*	234	7.31
*Canavalia gladiata* (sword bean)	41	1.28
*Glycine max* (soybean)	15	0.47
*P. mirabilis*	12	0.38
*Pseudomonas aeruginosa*	10	0.31
*Proteus vulgaris*	7	0.22
*Staphylococcus saprophyticus*	1	0.03
total	3200	

Retrieval from the ChEMBL 28 database was carried out by querying the corresponding SQLite database for all data with accession = P07374, which is the UniProt ID for *C. ensiformis* (Jack bean) urease. Additionally, only confidence scores equal to 8 or 9 were accepted and “*assay_type*” was required to be equal to “B” (binding assay). The structures for ChEMBL compounds were obtained as SMILES (as provided in the database). Additionally, we retrieved the literature (articles and patents) not covered by ChEMBL, and the structures from these sources were obtained through manual sketching of two-dimensional (2D) images of structures, which were then converted to SMILES, or from IUPAC-to-SMILES conversion using OPSIN.^17^

For the final data set, only IC_50_ and *K*_i_ values were used. To allow better comparability between assays, we normalized all activity values by dividing them by the activity of the control in the assay they originated from. Control compounds for Jack bean urease assays are typically thiourea or, more rarely, acetohydroxamic acid (both in the low μM range of activity). In a very small portion of the publications where a control activity was not reported, a control value of 20 μM was used for the normalization as this corresponds to a typical IC_50_ of thiourea. The data set was then divided into two classes of activity (actives and inactives) based on a normalized activity cutoff. This cutoff was defined by a rule that compounds with activity below that of the control are deemed active. As a result, activity ratios <1 were considered active, or otherwise deemed inactive. Finally, in cases where no IC_50_ was provided but the compounds were described as inactive, an excess value of 1000 μM was attributed.

All metal complexes and mixtures were removed, and SMILES were standardized and cleaned using the structure preparation library MolVS 0.1.1 (https://molvs.readthedocs.io/en/latest/) implemented in Python. The standardized SMILES were converted into InChIKeys, which were used to remove duplicates (keeping the most potent activity during deduplication). The final urease inhibitors data set was composed of 3200 molecules. All handling of the data was carried out in Jupyter Notebook using RDKit 2020.03.3, Pandas 0.24.1, and NumPy 1.15.4 (python libraries), integrated in Python 3.7.4. Whenever applicable, all further calculations used the standardized SMILES.

Lastly, 2D molecular descriptors were calculated using RDKit, which include drug-likeness/lead-likeness rules, water solubility, and polar surface area, among other descriptors relevant in medicinal chemistry.

### Structural Clustering

The SMILES of the compounds were converted to Morgan Fingerprints in RDKit using the *GetMorganFingerprintAsBitVect* function (radius = 2, bits = 1024), which were then used to cluster the data set using a hierarchical agglomerative clustering technique. To do so, we used the *AgglomerativeClustering* function implemented in scikit-learn 0.23.2.^18^ This work has two instances of clustering:

(a) “Spontaneous” clustering, where clusters were let to spontaneously assemble with only a constraint of shared similarity. To achieve this, we set the affinity parameter to “precomputed”, accompanied by the use of a precomputed Tanimoto distance matrix for all-vs-all compounds, and linkage = complete. This was done exclusively to assess raw diversity within the data set, which was quantified as the number of clusters produced.
(b) Clustering to build “structural families”, where the number of clusters was limited to allow a feasible manual analysis of their content. This was carried out to produce clusters from which we drew insights regarding the relationship between chemical families and activity. In this type of clustering, we tested the *n*_clusters parameter to a number ranging between 20 and 80, with all other parameters used as default (affinity = “euclidean” and linkage = “ward”). The optimal number of clusters was manually selected through inspection of cluster size (number of compounds) and cluster diversity (measured with minimum and mean intracluster similarity). A total of 50 clusters was ultimately selected as this offered a good compromise between a sufficiently tractable number of clusters and plenty of clusters with moderate-to-high intracluster similarity. Each of these clusters was submitted to a maximum common substructure (MCS) analysis using the *FindMCS* function in RDKit (setting *ringMatchesRingOnly* to True, to ensure ring atoms are only matched to ring atoms when comparing different structures). The MCS of a cluster is the largest substructure shared by all molecules in a given cluster, or in practical terms, the common scaffold. We report the minimum and median intracluster similarity which refers to the minimum and median values of similarities between every two compounds in a given cluster.

### Chemical Space Visualization

Visual clustering was also performed using the t-SNE implementation in scikit-learn (https://scikit-learn.org/stable/modules/generated/sklearn.manifold.TSNE.html), which compressed the 1024 original dimensions into two dimensions. We ran t-SNE under default parameters, and no dimensionality reduction was applied prior to t-SNE fitting. This tool is highly effective in taking relative distances within a certain neighborhood in the high-dimensional space and conserving them in the new low-dimensional matrix.^19^ This method is preferred for the purpose of visualizing chemical space (represented as Morgan fingerprints) when compared to other regularly used methods such as principal component analysis (PCA) or multidimensional scaling (MDS) as it focuses on preserving small distances in the data, whereas PCA and MDS focus on preserving large distances.^19^ This is particularly useful to produce visual clusters within a data set and, as a result, t-SNE’s clusters often correlate to structural differences.

### Scaffold Analysis and Activity Cliffs

The two classes, actives and inactives, were also characterized in terms of Murcko Scaffolds,^20^ calculated for each compound with RDKit, typically also used to gauge diversity. Additionally, to explore interesting transformations between compounds, we extracted all activity cliffs in the data set (*i.e.*, compounds with high similarity and yet a high shift in activity). A complete list of activity cliffs was obtained by running a similarity search of each active against all inactives, and *vice versa*. Pairs with a Tanimoto coefficient (T_c_) above 0.6 were deemed activity cliffs. A threshold of 0.5–0.55 is commonly used for activity cliff analyses,^21^ but in this work, a more stringent threshold of 0.6 was used.

### Compound Similarity

All similarity values mentioned throughout this work refer to the Tanimoto coefficient (or Tanimoto similarity) calculated over Morgan fingerprints (radius = 2, bits = 1024), which spans between 0 (minimum similarity) and 1 (maximum similarity).

### Extraction of Structural Rules and Meaningful Features that Drive Activity

Decision tree-based anchors were calculated to extract meaningful structural rules associated with activity using the *DecisionTreeClassifier* function in scikit-learn (https://scikit-learn.org/stable/modules/generated/sklearn.tree.DecisionTreeClassifier.html) paired with the Anchors (or scoped rules) method^22^ through the use of the anchor-exp Python library. To do so we first trained a decision tree model where, to ensure a sufficiently general tree, we capped the maximum depth at 10; all other parameters were used as defaults. This model was created for pattern extraction purposes only, and neither was it used to derive any predictions nor should it be interpreted as a predictive model. After the decision tree was trained, it was submitted to an anchor-generation procedure, adapting the jupyter notebook in the anchor-exp’s GitHub repository (https://github.com/marcotcr/anchor/blob/master/notebooks/Anchor%20on%20tabular%20data.ipynb). A precision threshold of 0.80 was used (applied to AnchorTabularExplainer).

As a complement to the rules, we also calculated the feature interaction scores (which measure feature importance) using the iml R package (https://cran.r-project.org/web/packages/iml/). This was done by adapting an R workflow in the iml GitHub repository (https://github.com/christophM/iml/blob/main/notebooks/tutorial-intro.ipynb) integrating (1) the iml package to obtain the feature interaction analysis and (2) the mlr and caret R packages for machine learning.

## Results and Discussion

### Overview of Temporal Trends in the Development of Urease Inhibitors

Starting a new drug development campaign and assessing the activity of compounds is a hard endeavor. Thus, having a basic understanding of the chemical space already tested provides useful knowledge on what structural features impact binding to a target and can guide a more efficient discovery of new hit compounds. More importantly, knowing what has already been done prevents accidentally investing in compounds or scaffolds previously explored, which already yielded poor results. To this end, we wanted to carry out a survey of all researched urease inhibitors and perform an in-depth analysis of active and inactive compounds and their underlying trends, as no such study exists.

To do so, we carried out extensive literature and patent review to assemble a data set containing inhibitors tested against ureases of nine different species covering a total of 238 references. Even though different ureases are targeted for different applications, due to the highly conserved active site among different species,^14,15^ it is likely that compounds tested against one particular urease will also be active against ureases from the other species. Thus, aggregating compounds tested in different ureases provides a more complete picture of which compounds have been developed for urease inhibition in the last few decades. Furthermore, this is also useful for the development of urease inhibitors since the assessment of translatability into other important ureases such as *H. pylori* and *P. mirabilis* is highly desirable. Nonetheless, even though many species are included, Jack bean (*C. ensiformis*) urease is overwhelmingly the most used in *in vitro* models (see Table 1). After a full curation process, we obtained a final data set of 3200 small molecules that spanned a large range of activities from subnanomolar to high millimolar concentrations and was composed of 1633 inactive molecules and 1567 active molecules.

**Figure 1:**
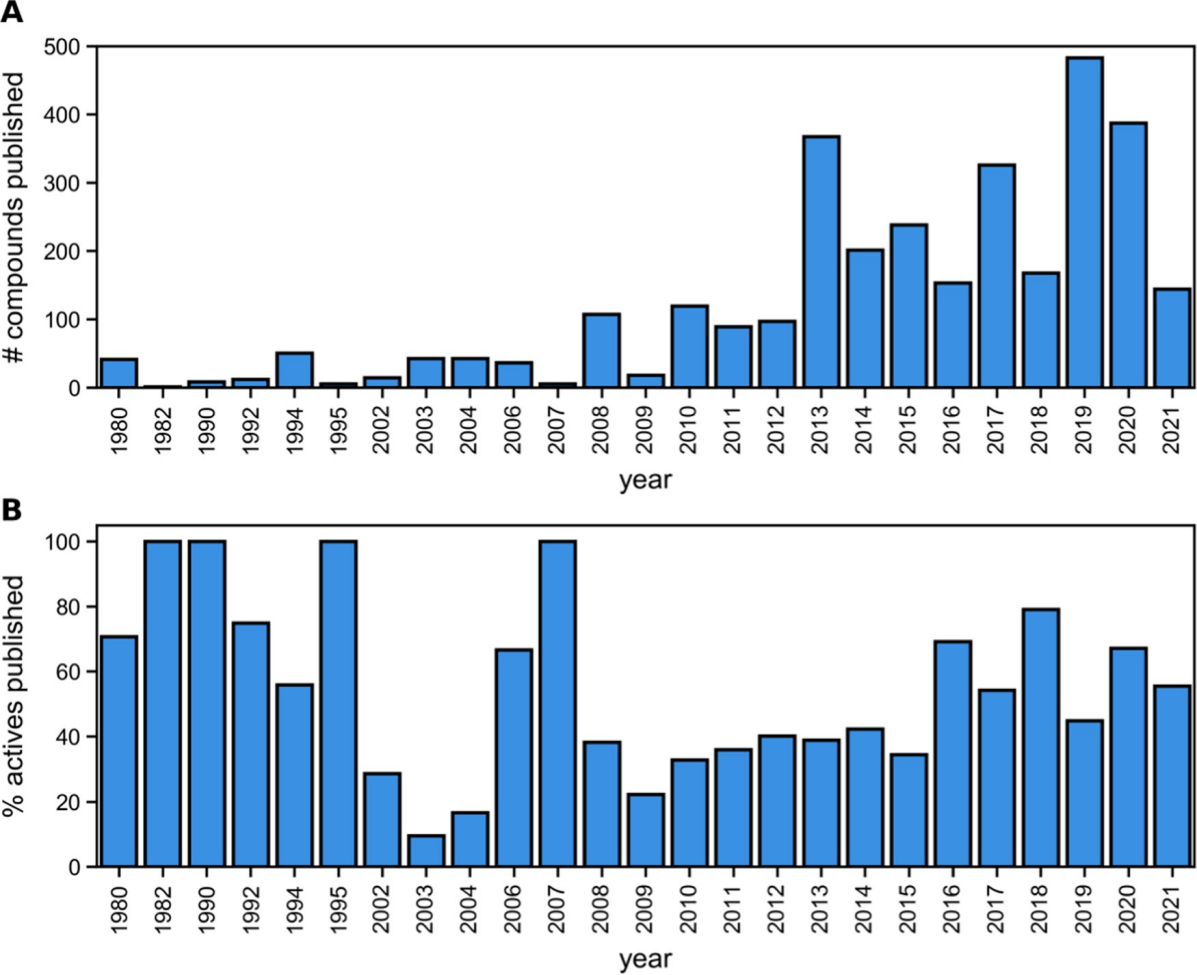
(A) Number of compounds published throughout the years and (B) percentage of active molecules published. We can observe an increasing trend in the relative active molecules found from 2009 onward and a decreasing trend in earlier decades.

Looking at the trend of compounds published throughout the last few decades (Figure 1), we observed that 2013 and 2019 are the two most prolific years in terms of the raw number of reported compounds. However, only relatively small portions of the compounds were active (36 and 55%, respectively). Another observation is that up to 2008 relatively small amounts of compounds were introduced with varying efficiencies, and from 2009 onward, there is a trend of rising efficiency in the discovery of new hits (*i.e.*, increased proportion of actives discovered). Interestingly, there also seems to be a small trend of increasing the average molecular weight of the compounds, but the lipophilicity, topological polar surface area (TPSA), and the number of hydrogen-bond donors and acceptors seem to remain stable (see Figure S1).

### Distribution of Physicochemical Properties and Drug-Likeness Analysis

**Figure 2:**
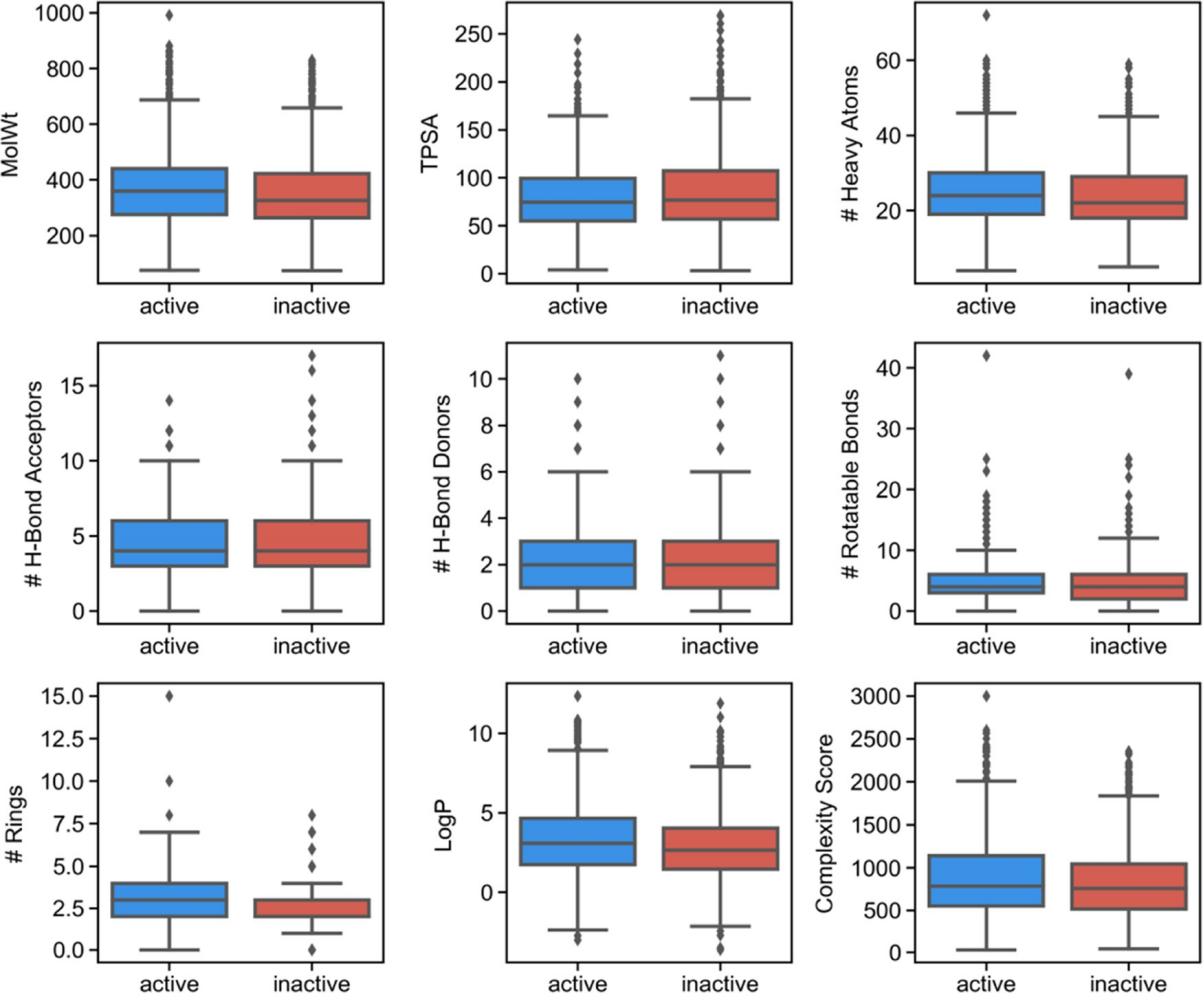
Comparison of key physicochemical features in active (blue) *versus* inactive (red) compounds. No differences can be observed between both classes for the different properties. The complexity score corresponds to the BertzCT descriptor in RDKit, which is calculated based on the bonding complexity and the complexity of distribution of heteroatoms and developed by Bertz.^23^

Next, we assessed how active and inactive compounds compare, with respect to common physicochemical features, as well as how they are positioned with respect to common drug-likeness and other medicinal chemistry filters. As depicted in Figure 2, the distribution of active and inactive compounds in nine typically assessed physicochemical features revealed no marked differences between the two classes. This suggests that urease inhibition is not determined by or correlated to a particular feature, but, as we will argue in the following sections, it is rather determined by small changes or the presence of certain scaffolds and/or substructures.

Furthermore, both the molecular weight and log *P* do not seem to be having a direct effect on the activity, which is generally a consequence of increasing the lipophilicity for the sole purpose of increasing the activity (*i.e.*, unspecific binding). Furthermore, even for the most potent compounds (activity lower than 100 nM) corresponding to the top 136 molecules, no significant differences were observed in the distribution other than having a slight tendency for fewer hydrogen-bond acceptor groups (Figure S2).

**Figure 3:**
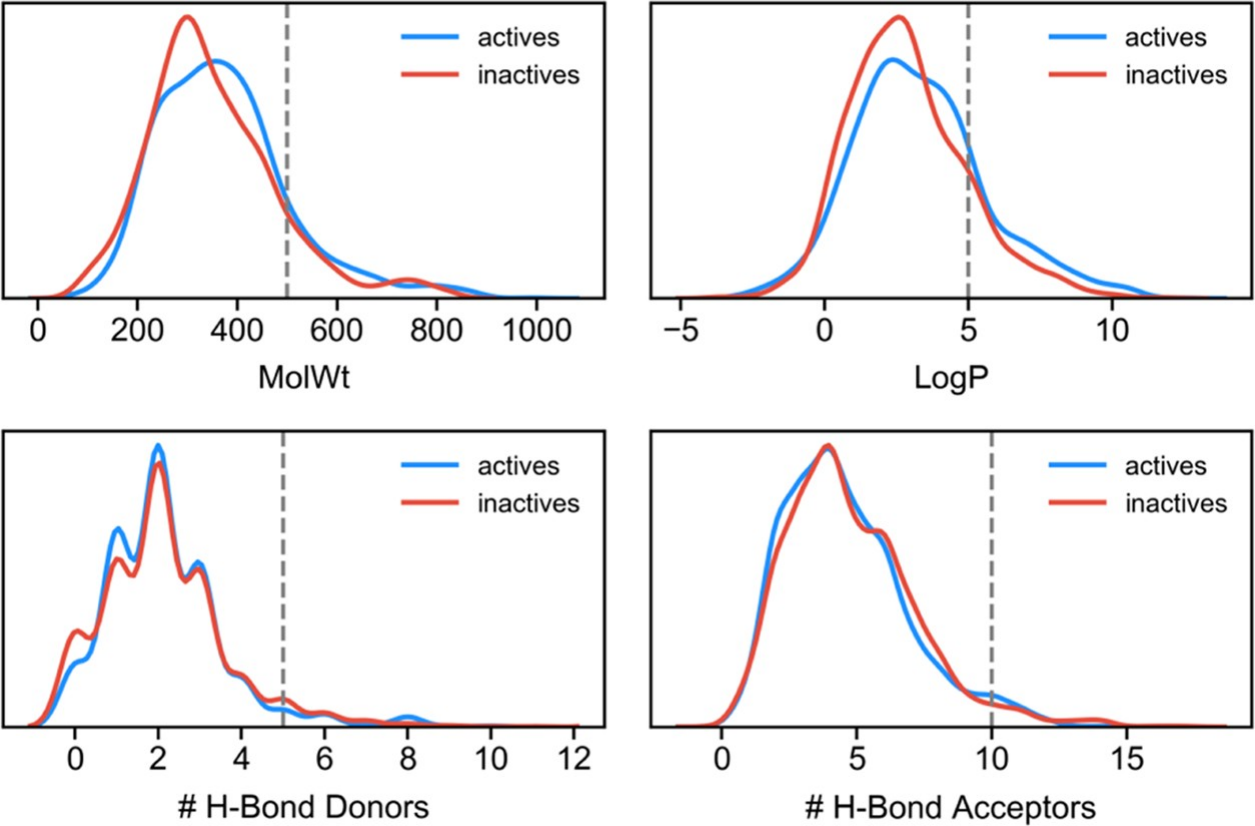
Distribution of urease inhibitors (blue) and noninhibitors (red) with respect to the four rule-of-5 descriptors: molecular weight, log *P*, and the number of hydrogen-bond donors (hbd) and acceptors (hba). The gray dashed line indicates the maximum value allowed for each descriptor. Both classes show an overlapping distribution.

Understanding how these inhibitors fall with respect to various rules of medicinal chemistry and lead- or drug-likeness may indicate how these rules have influenced development. Among them, Lipinski’s rule-of-5 is used to evaluate such drug-likeness of a compound. Applying the criteria of this rule to both active and inactive compounds showed that most of the compounds in both classes are within the constraints of the rule (Figure 3). Nonetheless, there was still a significant number of compounds that fell outside this filter, specifically breaking the limits of molecular weight and/or log *P*. Overall, there was no difference between active and inactive classes, which indicates that the activity is not being driven by the so-called “molecular obesity” effect. These results were also observed for the top-ranked active compounds as well (Figure S2), and therefore, these rules were shown not to be discriminatory of activity. Overall, the distribution of active and inactive compounds closely overlapping is likely an indicator that the rule-of-5 may have influenced the selection of compounds that were being designed and tested over the last years. More importantly, these observations indicate that filtering the compounds using the rule-of-5 is not helpful in ensuring better hits as it would remove as many active as inactive compounds.

### Presence of Pan-Assay Interference Compounds (PAINS) and Compounds Bearing Unwanted Functional Groups (Brenk Filter)

Another typical filter widely used by researchers to select compounds for testing is the PAINS filter.^24^ This flags a number of functional groups that have been historically associated with false actives in different assays,^24^ and it would therefore be useful to inspect for the occurrence of these substructures in the urease inhibitor data set. The analysis of the urease inhibitor data set revealed that only 17.1% of the total amount of compounds did not pass the PAINS filter, with 15.1% of the total active compounds bearing PAINS structures.

Additionally, filtering compounds for the presence of unwanted moieties (*i.e.*, substructures associated with toxicity, high reactivity, etc.) is also common practice, as is evident by the implementation of a “clean” subset of ZINC, for example. In this regard, we screened our data set for the presence of such unwanted moieties as listed by Brenk et al.^25^ (i.e., Brenk filter) and observed that a significant number of compounds were flagged for removal (Table 2). Moreover, in most cases, these were more frequent in the active molecules except for Michael acceptors, coumarin, and catechol substructures. Curiously, the most affected families of compounds were those often associated with activity against urease, including the thioureas, imines, and hydroxamic acids, which usually bind to the nickel ions of the active site. Another common flag was the presence of Michael acceptors, which coincided with strategies for urease inhibitors that bind to the cysteine in the mobile flap and are therefore the reason for the loss of specificity.

**Table 2:** Most Common Unwanted Groups (as Defined by Brenk et al.^25^) Found among the Urease Inhibitor Data Set, and the Corresponding Number of Compounds Containing Them

substructure type	number of compounds	active molecules
oxygen–nitrogen single bond	1164	648
thiocarbonyl	898	516
imine	557	341
Michael acceptor	448	145
hydroxamic acid	324	155
nitro	311	151
aliphatic long chain	172	101
coumarin	175	61
catechol	150	32
thiol	85	69

If both filters (PAINS and Brenk) had been applied early on, this would have resulted in only 227 molecules (14.5%) being selected for further studies. Among this set of selected molecules, the 10 most active compounds are mostly composed of phosphorodiamidates and phosphoramides, with 4-chlorophenylphosphorodiamidate (4Cl-PPD) being the most active compound. Additionally, two interesting compounds would also have been kept, such as a sulfonamide analogue of sulfadiazine^26^ (among the top 3) and 3-(3-methylphenyl)-1-(1-phenylethyl)urea. The latter has nanomolar-range activity against Jack bean urease, but it has also shown activity against β-glucuronidase and phosphodiesterases,^27^ which could lead to off-target toxicity. Overall, these different scaffolds could be interesting to pursue the development of novel compounds.

It should be noted however that even though these filters can be employed as valuable starting points for compound development, one should consider that they come with their own caveats and can exclude useful compounds from being tested. Indeed, with regard to urease inhibition, we observed that most active compounds would have been excluded.

### Chemical Space Visualization and Chemical Diversity

**Figure 4:**
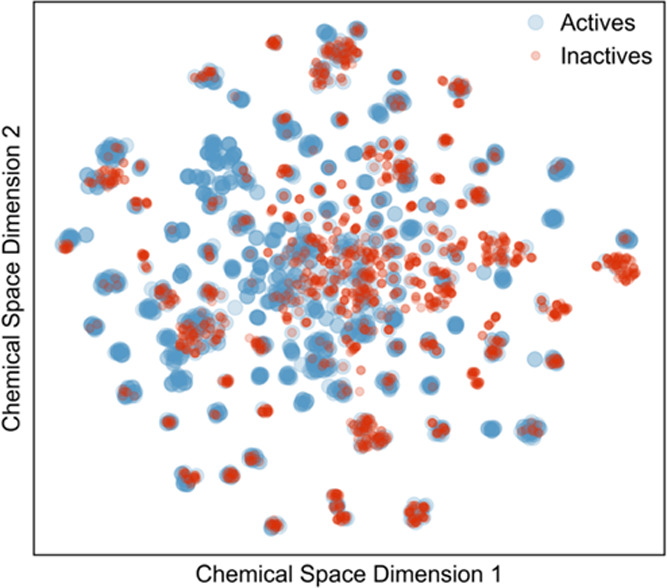
t-SNE distribution of chemical diversity of both active (blue) and inactive (red) compounds calculated from t-SNE. Active compounds were shown to have higher diversity.

Visualizing the chemical space can be useful to gauge the chemical diversity in a set of molecules. For instance, it can be useful to visualize if active and inactive compounds occupy the same chemical space. The chemical space distribution for urease inhibitors was plotted using t-SNE and is depicted in Figure 4. A significant number of clusters was observed, which attests to the broad chemical space and diversity explored for urease inhibition. However, active compounds have a higher chemical diversity since they occupy more clusters, as shown in Figure 4. In fact, inactive compounds exist almost exclusively in overlap with active compounds, while active compounds were found in regions that do not have inactive compounds. This is likely a result of compound optimization, which tends to be applied to prior actives based on SAR insights derived from experiments, and can also be derived from privileged scaffolds against other metalloenzymes.

To understand and quantify the overall diversity in our data set, we performed “spontaneous” clustering (see Methods section), where we set a maximum Tanimoto distance (1-T_c_) threshold of 0.6. This allows one to spontaneously generate clusters with considerable intracluster similarity, and the number of obtained clusters is, therefore, an indication of the chemical diversity. Using this analysis, a total of 417 clusters were obtained, which is a significant number compared to the total of 3200 compounds. Among all clusters, 109 were exclusively composed of actives but small in size (median cluster size of 2 compounds, and the largest of these actives-exclusive clusters had 39 compounds). Additionally, 90% of all 417 clusters had 20 or less compounds. Similar to the t-SNE analysis, this indicates that this data set covers considerable chemical diversity. It is important to note that this “spontaneous” clustering analysis was *exclusively* meant to probe the overall diversity in chemical space.

### Clustering Analysis to Assess Trends of Activity and Overall Diversity

Medicinal chemists tend to perceive chemical space diversity as the variety of known families, where each family has a particular scaffold. Although practical, this approach can be overly simplistic and biased by how chemists define a scaffold. In practice, a group of compounds may share multiple substructures, all of which contribute to urease activity and may result from hybrids of different “classical” scaffolds. In fact, there are several reports of hybrid compounds in an attempt to increase the activity against urease.^28−30^ Therefore, we used a more data-driven approach to characterize families of compounds. To do so, we employed hierarchical clustering (a manually optimized version) to the whole data set.

Since the number of spontaneous clusters was too large to be effectively analyzed, here the initial number of clusters was manually tuned to balance between clusters that are too small and more cohesive (higher intracluster similarity) and clusters that are too large and less cohesive. Selecting 50 clusters resulted in a good balance between size and cluster cohesion (see Figure S3), with the smallest cluster size (cluster 37) being 13 compounds. A total of 11 clusters showed high cohesion (*i.e.*, minimum intracluster similarity ≥0.5), and most of them are among the smallest clusters. This was expected considering the diversity of chemical space identified, however, among these clusters, 25 and 39 showed a considerably large size.

After the 50 clusters were obtained, the maximum common substructure (MCS) for each cluster was determined to find the common scaffold, which might not be reported as a known family *per se* but is still relevant to consider. Therefore, instead of guiding the analysis using predetermined scaffolds, we used data-driven scaffolds (*i.e.*, the MCSs from the clusters). This way, compounds in different reports that focus on different subgroups can be considered together if they share sufficient similarity (causing them to belong to the same cluster). This allows uncovering broader and more meaningful positive and negative structural modifications to urease inhibitors.

Even though there were a considerable number of clusters, they showed a high median intracluster similarity (majority above 0.5) (Figure S3 and Table S1). Therefore, the scaffolds derived from MCSs are more likely to be a better representation of their cluster. Nevertheless, there are multiple large clusters with low median intracluster similarity, which corroborates the earlier observation of the high diversity of compounds screened against ureases. A summary of all clusters, their statistics, and MCS is shown in Table S1, and a distribution plot showing intracluster similarity for each cluster is shown in Figure S3.

#### Analysis of Large Clusters

The clusters with the largest numbers of compounds can reveal trends of substructures that have been under heavy focus by the scientific community. The biggest cluster observed (cluster 7) contained 674 molecules (21% of the total data set), with a median intracluster similarity of 0.2, and 49.4% of its molecules being active (Figure S3). This is therefore a very diverse cluster, and even though there was no single common scaffold, this cluster contained small-molecular-weight molecules with multiple scaffolds that structurally resemble urease’s native substrate (urea). These compounds included phenylsulfonamides, phenylureas, benzylhydrazines, phenylphosphoramides, phenylphosphorodiamidates, and benzisoselenazoles. Such scaffolds are usually associated with activity, and not surprisingly, cluster 7 contained some of the most potent compounds found (IC_50_ as low as the picomolar range of activity).

Similarly, the second-largest cluster (cluster 20) contained 175 molecules with a median intracluster similarity of 0.23 with no common scaffold. This cluster was composed of phosphoramides, thiobarbituric acids, thiazolidines as well as other small fragments. Even though this cluster was mostly populated by inactive molecules (34.9% actives), the active molecules in this cluster reach the low nanomolar range of activities.

**Figure 5:**
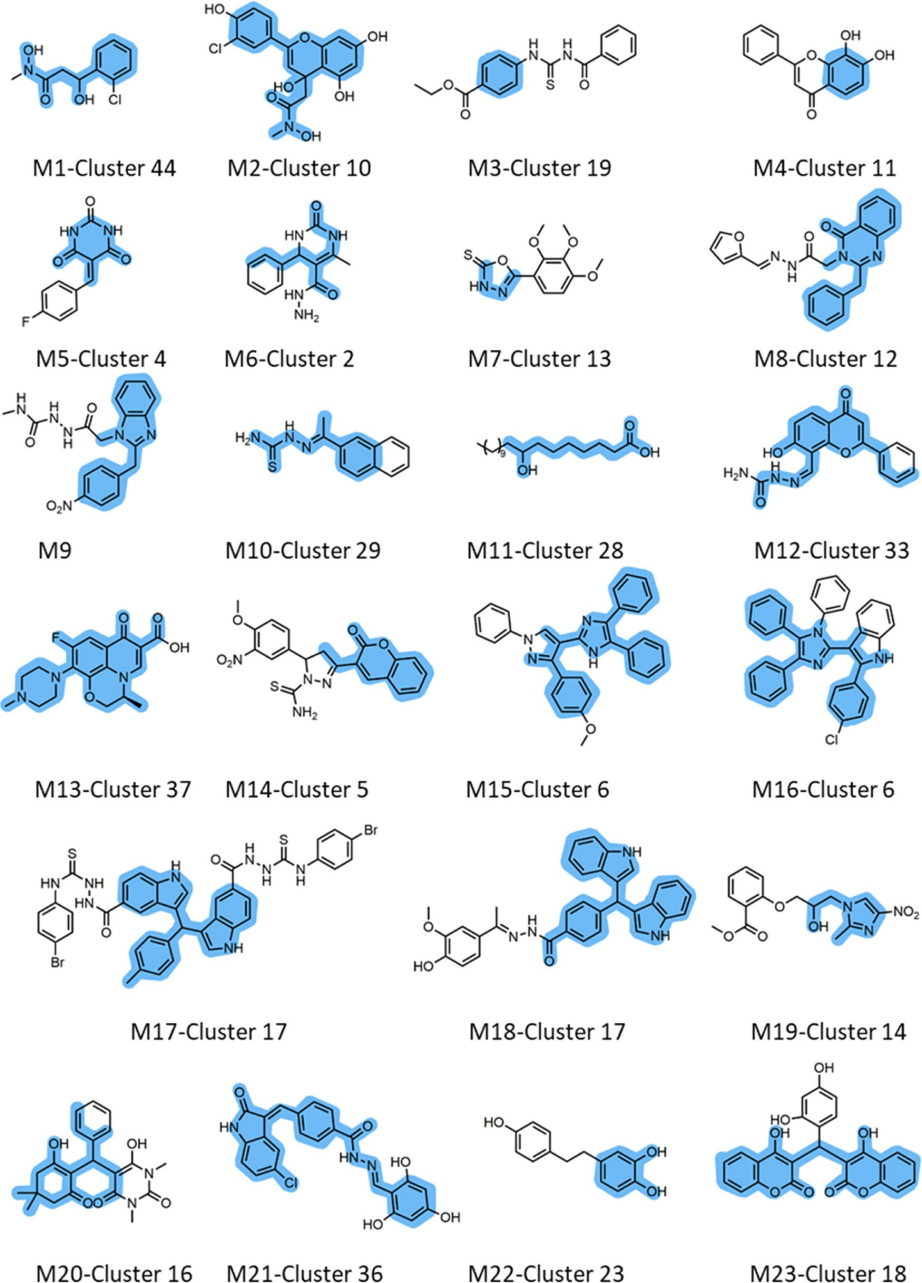
List of scaffolds (in blue) with significant variations in activity represented in different clusters, represented using exemplary molecules. The blue highlighted structure illustrates the common scaffold obtained through maximum common substructure decomposition for each cluster.

Surprisingly, the third biggest cluster (cluster 15), representing a total of 155 compounds with a median intracluster similarity of 0.37, showed an astounding 71% active molecules. This cluster had a higher similarity between its molecules, compared to the previous clusters (median 0.37 *versus* 0.23 and 0.20; see Figure S3 and Table S1), and its molecules share the hydroxamic acid group, which is a functional group associated with activity.^4^ The importance of clustering a diverse data set of compounds to evaluate meaningful patterns of activity is illustrated by the fact that cluster 44 (78 compounds) also has hydroxamic acid compounds (Figure 5, M.1.) but only contained 19.2% active molecules. In fact, the overall analysis of all hydroxamic acids in our database (306 molecules in total) showed that only 48.3% of the molecules were active. However, upon closer inspection, we realized that cluster 44 together with the flavonoid analogues bearing a methyl-hydroxamic acid originating from cluster 10 (50 molecules; Figure 5, M.2.) had only 20% of active molecules, making them the main culprit for an apparently low overall frequency of actives of 48.3% associated with the hydroxamic acid functional group. Therefore, this indicates that simply introducing groups of known activity as the sole strategy to increase activity may not be a good strategy to design actives.

The temporal trend analysis for clusters 15 and 44 (Figure S4) showed that, as would be expected from the popularity of the hydroxamic acid scaffold, the molecules in cluster 15 have been accumulating since the early 1980s up to 2020, whereas molecules in cluster 44 were only reported between 2010 and 2017. Running a similarity search between older molecules and the most recent molecules in cluster 44 revealed that many of the more recent molecules (55%) are the closest (and significantly similar, *T*_c_ > 0.47) to an older inactive compound (IC_50_ > 20 μM, considering thiourea’s activity as the cutoff). This is just another piece of evidence of the complex, nonlinear nature of compound derivatization in the search for actives. Note that all temporal trends for the various clusters are shown in Figure S4.

Another interesting cluster with a significant number of compounds (*N* = 140) was cluster 19, which contained 56.4% active molecules (Figure 5, M.3.). This cluster aggregated molecules with moderate similarity (minimum and median intracluster similarities of 0.24 and 0.51, respectively) and was represented by a variety of different *N*^1^-benzoyl,*N*^2^-aryl-thioureas with different substitutions. The activity of these compounds varied wildly, with the best compounds having inhibitory activity around 130 nM. This type of compound is typically classified as mixed-type inhibitors,^31^ and there seems to be an ideal size for activity (Figure S5). Further inspection of cluster 19’s content showed that symmetrical bis(benzoylthioureas) were associated with loss in activity. *N*^2^-aryl esters and sulfonamides were also found in these clusters and were all active compounds, even containing the most active compounds among the full data set. On the other hand, hydroxy and methoxy substitutions in this ring resulted in a loss of activity, seeing as only one hydroxy and two methoxy substitutions out of a total of 19 compounds with similar substitutions were active. Additionally, dimethoxy substitutions on the *N*^1^ ring were associated with activity. Interestingly, the closely structurally related phenyl-3-phenylpropanoyl thioureas in cluster 42 composed of 29 compounds (Table S1) showed 79.3% active molecules, but their activity was in the picomolar range, with the most potent being reported as a competitive inhibitor and two others were mixed inhibitors.^32^ Similarly, cluster 28 (Figure 5, M.11.) also contained *N*-acyl thioureas with long alkyl chains exhibiting activities in the low nanomolar range, but the most potent compounds were found to be noncompetitive inhibitors.^33^ This demonstrates that typical medicinal chemistry conventions of how we perceive compounds to belong to a given family can be misleading and data-driven analysis such as this one allows identifying a promising scaffold that offshoots from a larger group of seemingly fewer promising compounds.

Cluster 11 (Figure 5, M.4.) had a significant number of compounds (118) with a slightly larger median intracluster similarity of 0.31 and only 21.2% active molecules. This cluster corresponds to flavonoids, which are generally associated with low activity.^34^ Many flavonoids have been tested as they can be extracted from many plants but are known to be only moderate inhibitors of urease even though competitive inhibitors have been found. As discussed above, specific modifications seem to drive the activity rather than the scaffold itself, as shown by the addition of hydroxamic acid to the flavonoid scaffold that did not significantly improve their activity. For instance, there are 30 hydrazine-flavonoid hybrid compounds produced by one study, of which 90% were active. These were allocated to cluster 33 (Figure 5, M.12.), and as observed for cluster 11 (118 compounds, Figure 5, M.4.), this cluster overall contained only 21.2% actives. Similarly, cluster 10 (Figure 5, M.2.) also contained 50 flavonoid hybrid molecules, of which only 20% were active.

Barbiturates and thiobarbiturates have also been extensively studied due to their urea-bearing structure. However, barbituric and thiobarbituric acid analogues are also typically associated with moderate-to-low activity.^35−40^ These molecules were aggregated in cluster 4 (Figure 5, M.5.), which showed high median intracluster similarity (0.46) despite the relatively large size (*N* = 130), and low percentage of actives (34.6%). Various modifications of the phenyl ring have been tested, and extending the ring in the para-position seemed to increase the activity except when sulfonamides were used as linkers. On the other hand, closely related dihydropyrimidines found in cluster 2 (106 compounds with a mean intracluster similarity of 0.45) contained 55.7% active molecules (Figure 5, M.6.), showing that clustering could separate closely related compounds in a meaningful way that actually correlated better with activity. For the latter cluster, it was observed that higher potency was achieved with isatin > thiosemicarbazide > phenyl substitutions, as well as with a high number of substitutions of electron-withdrawing groups.

Finally, among the clusters with the largest number of compounds, cluster 13 with 117 compounds (Figure 5, M.7.) aggregated mostly a series of both oxadiazole and triazole thione analogues from different reports with a median intracluster similarity of 0.30, which was also associated with the poor ability to produce actives (only 27.4% actives).

#### Analysis of Clusters Mostly Populated by Actives

The clustering of urease inhibitors revealed 10 clusters with a total of 365 molecules, where each cluster had at least 80% active compounds (Table S1). These are likely the “safest bet” clusters from which to develop new compounds, considering their high rate of active molecules.

**Figure 6:**
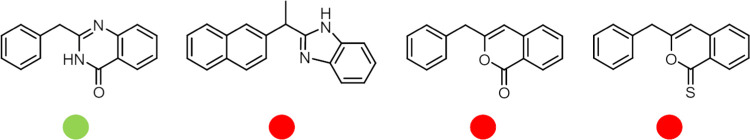
Active-enriched scaffolds from cluster 12 (green dot) *versus* three inactive-enriched scaffolds (red dot) structurally closely related.

Among them, cluster 12 (Figure 5, M.8.) was a highly cohesive cluster (minimum intracluster similarity of 0.48) that had a total of 40 molecules bearing the 2-benzyl-3,4-dihydroquinazolin-4-one scaffold, all of which were active compounds. Interestingly, while manually optimizing the total number of clusters, we noticed that in some cases another set of molecules with the 2-benzyl-1*H*-1,3-benzodiazole scaffold from another cluster (Figure 5, M.9.) was aggregated with this scaffold. In the final clustering setting used, these molecules were clustered in a different cluster of 88 molecules associated with high activity (95.5% active molecules) that represented a mix of scaffolds (median intracluster similarity of 0.27) of nitrogen-rich compounds. Nevertheless, this class of compounds had a common denominator with cluster 12, which can be observed by the high similarity between molecules in both clusters (i.e., M8 and M9). The fact that cluster 12 was entirely composed of actives shows that the 2-benzyl-3,4-dihydroquinazolin-4-one scaffold is a very promising starting point for drug development. However, closely related scaffolds such as 2-[1-(naphthalen-2-yl)ethyl]-1*H*-1,3-benzodiazole, 3-benzyl-1*H*-isochromen-1-one, and 3-benzyl-1*H*-isochromene-1-thione were found to be enriched with inactive compounds (Figure 6).

Cluster 29 (Figure 5, M.10.) represented a moderate number of molecules (*N* = 36), which was enriched with active compounds (88.9%) that shared the acetophenone thiosemicarbazone as a common scaffold. This is a group with high potential for binding to the Ni ions of the active site. Cluster 28 contained 45 compounds (86.7% actives), among which there were potent urease inhibitors (low nanomolar range), whose common substructure is a long 12-carbon chain with a carbonyl moiety (Figure 5, M.11.). This cluster also included the 1-acyl-3-arylthioureas that, despite being associated with potent inhibition and despite including the thiourea group, have been shown by others to be noncompetitive inhibitors of Jack bean urease (so we have two separate SARs in this group).^33,41^

Various other aromatic-rich compounds were also associated with a high number of active molecules such as in the case of cluster 37 (13 compounds, Figure 5, M.13.), populated with data from three different reports and showing a total of 92.3% active molecules. Cluster 5 (Figure 5, M.14.) was composed of a set of 27 coumarin analogues, of which 85.2% were active compounds. Interestingly, even bulky scaffolds based on imidazole–imidazole and imidazole–indole motifs showed high activity, as observed for cluster 6 (26 molecules) with 65.4% actives, some of which reached the nanomolar range activity^42,43^ (Figure 5, M.15. and M.16.). Similarly, another set of bulky compounds was found in cluster 17 (*N* = 33 compounds), among which 57% were active. This cluster had a symmetrical scaffold, and interestingly, when the scaffold is disubstituted, it showed 100% activity (Figure 5, M.17.), with activities ranging down to the nM scale. However, a single substitution on the middle ring of the scaffold rendered this scaffold inactive (0% active, Figure 5, M.18.).

#### Analysis of Clusters Least Populated by Actives

Several clusters were associated with low activity and may provide insights into scaffolds with a high risk of failure. Among the clusters with the least actives, cluster 14 had a series of 51 secnidazole analogues tested against *H. pylori* urease containing only 15.7% active compounds (Figure 5, M.19.). As discussed earlier, even though barbiturates and thiobarbiturates are known moderate inhibitors, the 29 compounds that make up cluster 16 (Figure 5, M.20.) were all inactive. Cluster 36 (Figure 5, M.21.) comprising isatin analogues was another cluster with only 10% active compounds (for a total of 19 compounds).

Cluster 23 (70 compounds, Figure 5, M.22.) contained only 11.4% actives, with the most potent activity being 1.5 μM. This cluster contained compounds bearing catechol and pyrogallol and methoxy groups. This class of compounds is characterized by their potential to covalently bind with the cysteine on the mobile flap helix, and thus their specificity and potency may be lower.^44−47^ Finally, cluster 18 (87 compounds, Figure 5, M.23.) showed 19.5% active molecules. This is a cluster with a large common substructure consisting of two fused rings connected by a single carbon linker. Regarding this cluster, we observed the presence of a phenyl ring connected to the linker, from which extends a thiadiazol amine, resulted in gained activity.

### Clustering the Most Potent Compounds to Find Interesting Common Substructures

**Figure 7:**
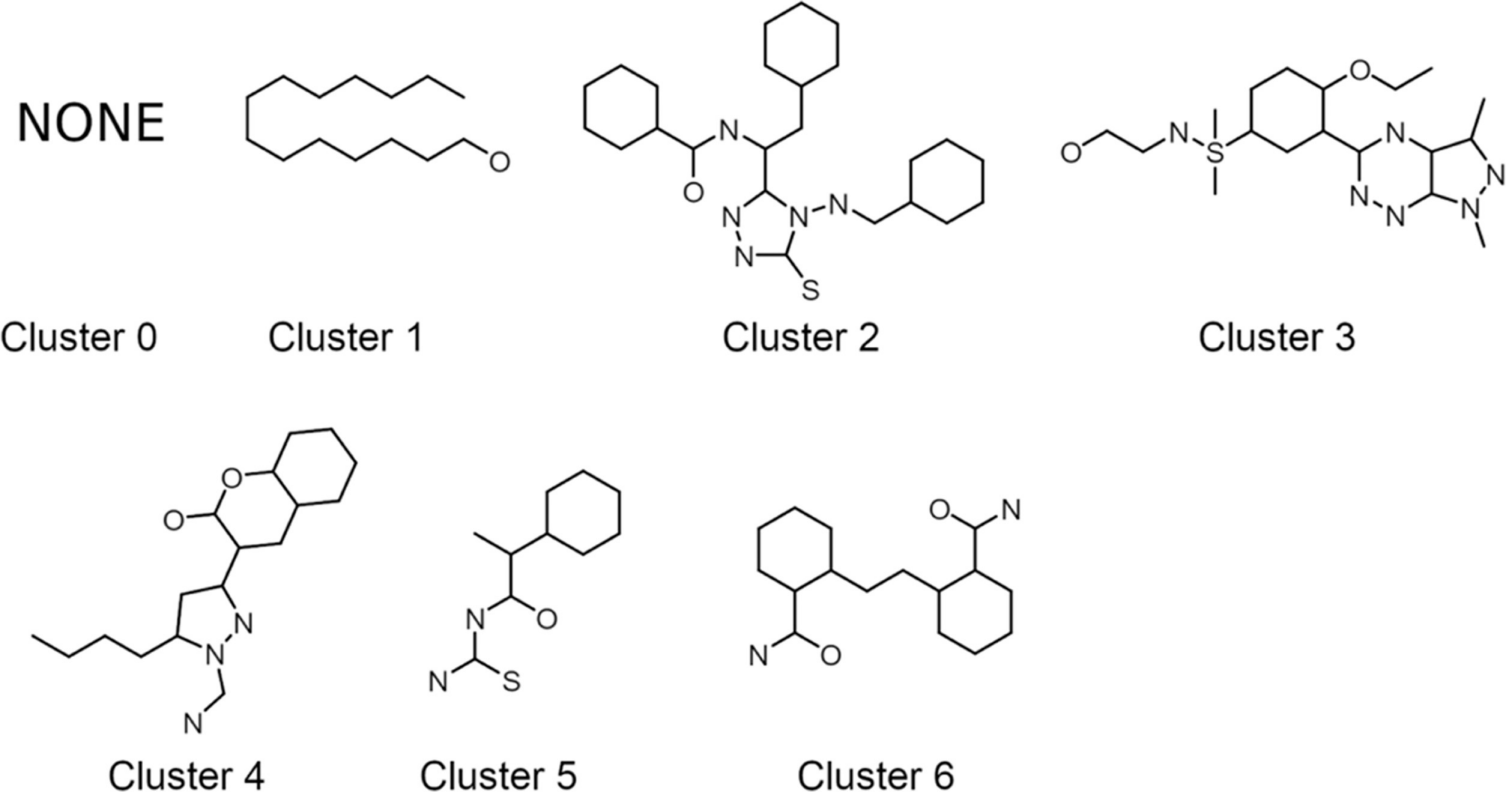
Maximum common substructures (MCSs) of the clusters formed by the top 100 most active molecules. The largest cluster (cluster 0, *N* = 57 compounds) did not show a common scaffold. As these structures are scaffolds, atom valences and bond types should not be considered as represented (only connectivity should be considered).

Applying the same clustering technique as before to only the top 100 most potent molecules showed a good diversity of scaffolds (Figure 7). The largest cluster (cluster 0) aggregated 57 compounds with no common scaffold and which corresponded to phosphoramidates and sulfonamides. The remaining clusters were smaller and corresponded to a variety of aromatic scaffolds and long-chain compounds as previously discussed (Figure 7).

### Physicochemical and Structural Rules that Drive Activity

**Figure 8:**
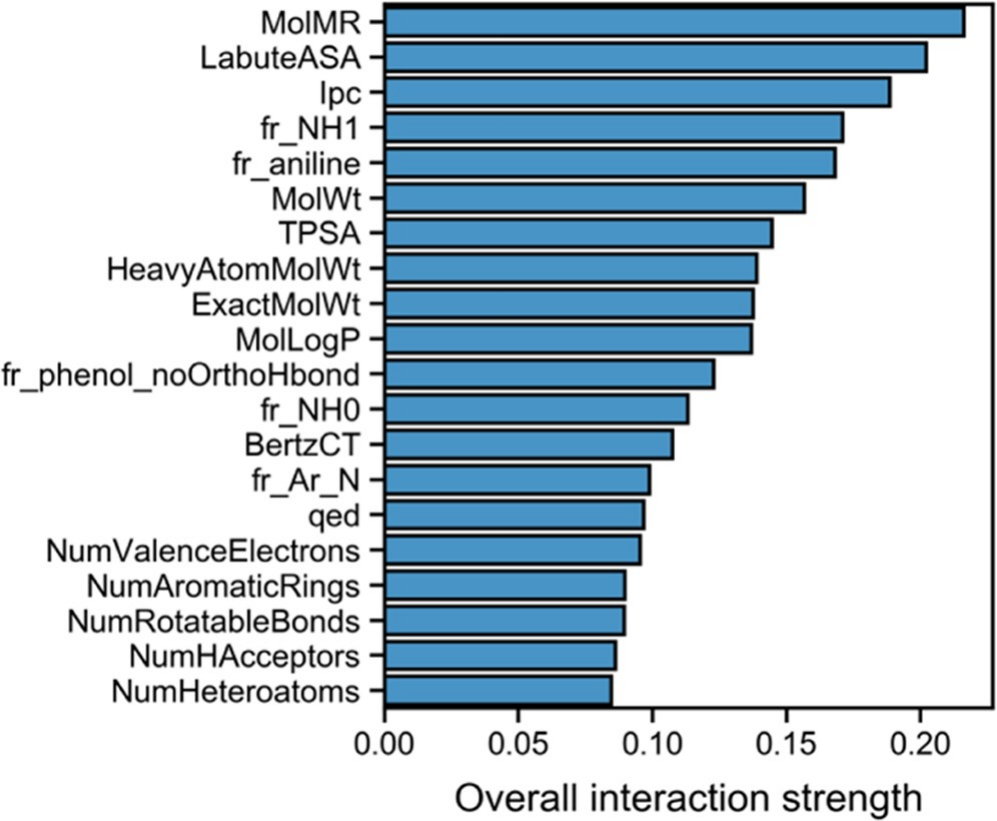
Feature interaction strength of the descriptors in the urease inhibitor decision tree model, which can be interpreted as a type of less biased feature importance.

To map how active and inactive compounds are differently distributed in chemical space in such a way that can be translated into rules or guidelines, we built a decision tree from all compounds available. Even though we limited the maximum depth to allow for more general rules, good memorization was still achieved (training accuracy = 90.8%), which means the decision tree was successfully able to separate actives and inactives in the data. The top 20 features, ranked in terms of importance (measured as the feature interaction score)^48^ within the decision tree, are shown in Figure 8.

Here, instead of using regular feature importance that is directly derived from the trained decision tree model, we used feature interaction. Feature interaction is defined by two or more features that, together, influence each other in the task of prediction.^48^ The interaction associated with a descriptor corresponds to the change in the predicted class that results from varying it in a feature pair. Figure 8 shows the cumulative effect of pairing each feature in the *Y*-axis with every other feature. Molar refractivity, measuring the real volume of molecules (MolMR), for instance, was revealed to be the feature that most influences other features in predicting the activity class of urease inhibitors. Curiously, this did not correspond to the top node in the decision tree (fr_NH1, number of secondary amines), which is expected given feature importance is context-dependent and simply evaluates the ability of a feature of separating the two classes received by a given node, whereas feature interaction is a global assessment where all features are considered with respect to all predictions. More details on the feature interaction metric can be found at https://christophm.github.io/interpretable-ml-book/interaction.html#interaction.

The high chemical diversity of this data set was once again evident by the production of a very large tree, with no particular branches associated with the classification of large subsets of compounds. To draw robust SAR rules, we calculated anchors using the anchor-exp python module, which were derived from the trained decision tree. Anchors are IF-THEN rules, which can be interpreted as sufficient conditions for a given activity outcome.^22^ Anchors are derived through a model-agnostic approach, using the recursive search for candidate rules that explain the classification of a given compound and perturbing the remaining features not covered by that rule by replacing them with features from another compound. These perturbed instances become the neighbors of the compound under focus and serve to probe how important a feature is for a given activity outcome. Applying this approach to our full data set, a total of 1185 anchors (rules) were obtained, of which we highlight the top 10 rules in terms of coverage, as shown in Table 3. Contrary to rules derived from the decision tree model trained, these anchors are much simpler (rules from the decision tree were often more than 10 parameters long), which improves interpretability and application to compound design/selection. However, it should be noted that anchors provide local SARs limited to a specific region of the descriptor space and are not exhaustive (*i.e.*, covering the entire chemical space of the full urease inhibitors data set).

**Table 3:** General Physicochemical and Structural Anchors (Rules) to Predict Urease Inhibition Activity^a^

rule #	rule	*N*_actives_	*N*_inactives_	*N*	Pre (%)	activity class
1	fr_NH1 = 0; fr_Ar_OH > 0	15	240	255	94.1	inactives
2	fr_NH1 = 0; fr_N_O > 0	26	122	148	82.4	inactives
3	fr_NH1 = 0; fr_NH0 = 0	6	138	144	95.8	inactives
4	fr_NH1 = 0; fr_Ar_N = 0	5	55	60	91.7	inactives
5	fr_aryl_methyl > 0; fr_hdrzine > 0	59	0	59	100.0	actives
6	fr_aryl_methyl > 0; fr_sulfonamd > 0	40	2	42	95.2	actives
7	fr_aryl_methyl > 0; fr_SH > 0	32	6	38	84.2	actives
8	NumAromaticRings ≤ 3; fr_Ar_OH > 0	1	31	32	96.9	inactives
9	fr_NH1 = 0; fr_Al_OH > 0	1	29	30	96.7	inactives
10	fr_NH1 ≤ 1; fr_Al_OH > 0	6	19	25	76.0	inactives

a *N*_actives_ and *N*_inactives_ reported refer to the compounds that are associated with a particular IF-THEN rule that defines each anchor.

In many of the top 10 rules, amine group count (fr_NH1 and fr_NH0) appeared as one of the descriptors (and sometimes even as the only type of descriptor, such as in rule). Curiously, the presence of amine groups was a descriptor only present in rules for inactive compounds. The largest rule for inactives covers a relatively large amount of compounds (*N* = 255, 8% of the full data set) and also with a very high precision (94.1%). This rule states that the absence of secondary amines (fr_NH1) along with the presence of at least one aromatic hydroxyl group often leads to inactive compounds. The following three rules with the largest amount of compounds also define inactives, and all require absent secondary amines, accompanied by the presence of hydroxylamine groups or the absence of tertiary amines or aromatic nitrogens. The largest rules that define actives (rules 5–7) cover fewer compounds each (*N* = 38–59) but still with a large precision (>84%). All three rules state that compounds must have at least one aryl methyl site amenable to hydroxylation, accompanied by at least one hydrazine (fr_hdrzine), sulfonamine (fr_sulfonamd), or thiol group (fr_SH).

Interestingly, each of these rules covered a diverse set of compounds, which is another evidence of the nuance in the molecular determinants of activity, which goes beyond selecting a given family or scaffold to produce active compounds. Rule 1 (the largest inactives rule) covers compounds from nine clusters (7, 11, 15, 16, 18, 20, 23, 40, 47) and rule 5 (the largest actives rule) covers compounds from 10 clusters (0, 2, 7, 8, 12, 17, 24, 42, 48, 49). This means that the molecules defined by each anchor are structurally diverse but still have a similar physicochemical and structural profile, which separates them from compounds of the opposite class.

### Activity Cliffs, Substructural “Dead Ends”, and “Safe Bets” in Urease Inhibitors

**Figure 9:**
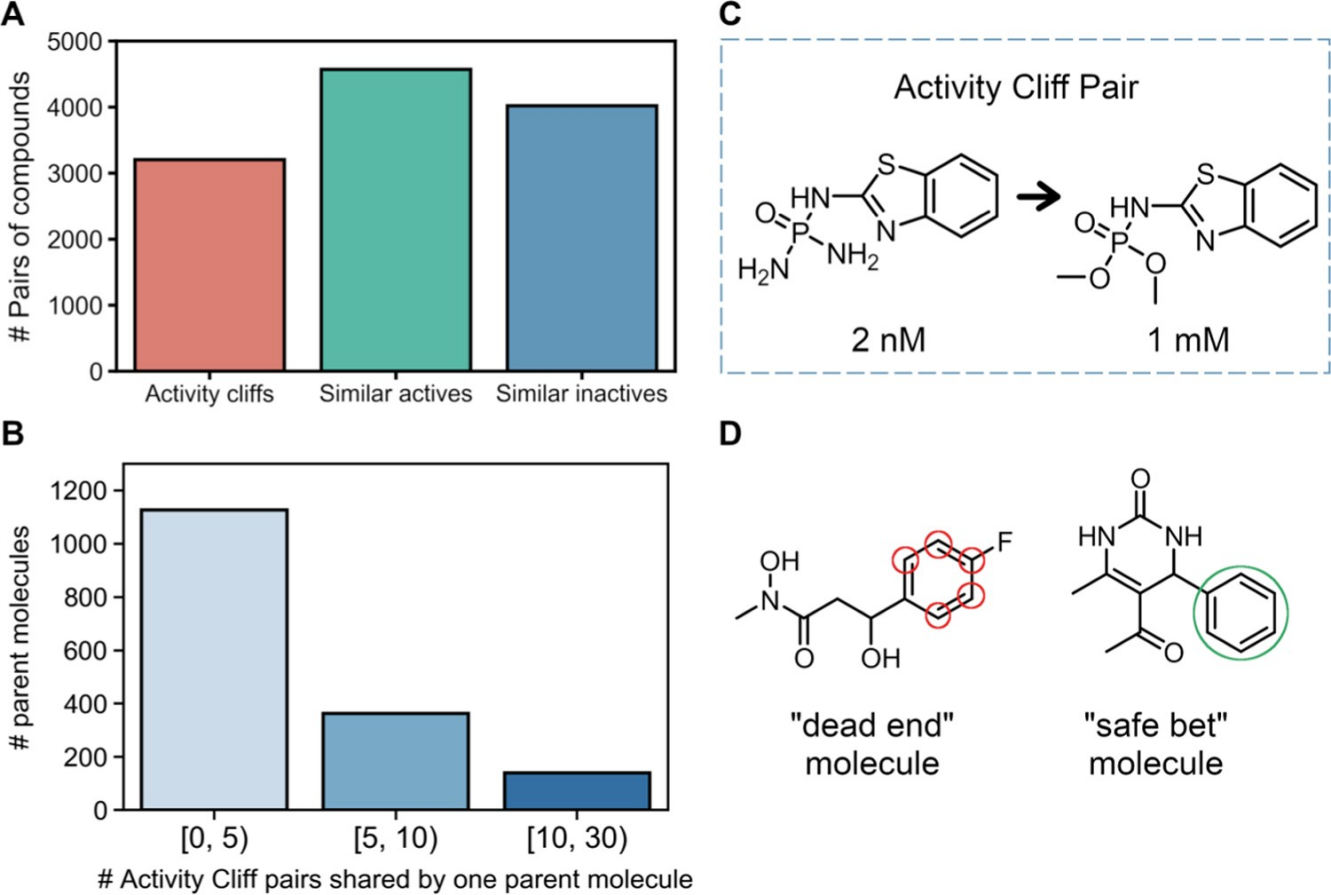
(A) Distribution of the different types of similar compounds in the data set. (B) Compounds that are involved in activity cliffs, and the corresponding distribution of activity cliff pairs per compound. (C) Example of an activity cliff pair where switching two amines for two methoxy groups produces a loss of activity by several orders of magnitude. (D) Examples of a “dead end” compound and a “safe bet” compound (additional information in the text). The red and green circles indicate positions where modifications led to loss and gain of activity, respectively.

One of the most useful and actionable pieces of information that can be drawn from all tested compounds against urease to date is what structural modifications (or R-groups) are consistently associated with loss or gain of activity. To analyze this, we identified all pairs of compounds that share considerable similarity (*T*_c_ > 0.6). These pairs were allocated to one of three possible groups: both compounds are active and share a similar structure (linear SAR pair), both compounds are inactive and share a similar structure (linear SAR pair), or the two compounds have different activities but share a similar structure (unpredictable SAR, *i.e.*, activity cliff). The distribution of the three categories is shown in Figure 9A, where a large portion of pairs was found to be activity cliffs. Activity cliffs are interesting to explore as they refer to portions of chemical space where structure and activity correlate unpredictably and small changes in structure lead to drastic changes in activity. An exemplary activity cliff pair is shown in Figure 9C. This analysis revealed that a rather large number of activity cliff pairs exist (*N* = 3206), corresponding to almost a third of all similar pairs of compounds. This indicates that navigating the urease inhibitors’ chemical space is quite challenging, which underlines the importance of inspecting all known compounds to learn which structures are prone to unwanted shifts in activity.

Despite the diverse chemical space, there is a significant number of pairs of compounds that are interesting to understand urease inhibition activity. The top 5 scaffolds (or cores) with the highest number of activity cliffs studied correspond to compounds from clusters 2, 10, 19, 36, and 44. Even though there is no way to fully avoid activity cliff compounds, there are cases where a given scaffold is frequently prone to the generation of activity cliffs. Therefore, we inspected the compounds most frequently implicated in an activity cliff pair. Figure 9B shows how many compounds are involved in how many activity cliffs, revealing that an astounding 139 compounds form activity cliffs pairs with 10 up to 30 compounds of the opposite activity class, *i.e.*, reference inactive compound + *N* paired actives or reference active compound + *N* paired inactives. The first scenario corresponds to what we call “safe bets”, and the second corresponds to “dead ends”.

A total of 85 active compounds fell into the dead-ends category (see Figure S6), as these form high-similarity pairs with 10–30 inactive compounds. For instance, a compound from cluster 44 was found in 20 pairs of activity cliffs (left-hand side of Figure 9D), where all activity-loss modifications occur in the aromatic ring, in the positions marked with a red circle. This compound is highly active (400 nM) but loses its activity up to multiple orders of magnitude when it undergoes small changes (switching a F for a Cl or switching the position of the F group from para to meta). Essentially, this means that modifying this compound (and perhaps similar compounds) at this location should be avoided or at least be done with care, using complementary structure-based studies to understand the role of these substituents in binding to the enzyme.

On the opposite side of the spectrum, there were 54 safe bet compounds, which consisted of inactive compounds whose modification always produced an active compound, for a variety of modifications (Figure S7). For instance, the inactive compound shown on the right-hand side of Figure 9D (IC_50_ of 1 mM) established 20 high-similarity pairs with active compounds, where all modifications that yielded those active compounds consisted of groups added to the phenyl ring or, in two instances, replacement of the phenyl by a thiophenyl group.

All safe-bet compounds originated from 14 clusters (2, 6, 8, 18, 19, 25, 26, 30, 32, 33, 35, 39, 42, 48) and all dead-end compounds originated from 19 clusters (1, 2, 10, 14, 17, 18, 19, 24, 25, 26, 27, 32, 36, 40, 41, 43, 44, 45, 47), which means that, in alignment with previous observations, this also underscores the complexity and diversity of the structure-activity landscape of urease inhibitors.

One should note that in this analysis, gain or loss of activity is not quantitative, and in some activity cliffs, compound activity may be close to the cutoff (*i.e.*, ratio over the control equal to 1). Therefore, some of the shifts in activity might be small in absolute terms. Still, a small but consistent loss or gain of activity in a given scaffold/core is still worth considering.

**Figure 10:**
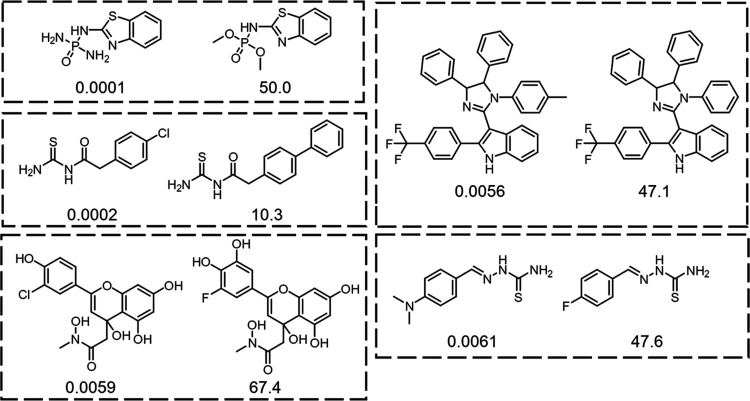
Most significant activity cliffs among urease inhibitors, labeled with the corresponding normalized activity (*i.e.*, activity/activity of assay control).

To complete this analysis, we looked at the most dramatic activity cliffs, and the top 5 pairs with the most dramatic shifts in activity are shown in Figure 10. Contrary to the previous examples, as these pairs are just isolated examples of a scaffold modification, they cannot be used to draw any general conclusions on which modifications lead to gain (or loss) in activity. Nevertheless, they are interesting to report.

### Overview of Well-Known Families of Urease Inhibitors: Are They as Effective in Yielding Actives as Expected?

Some chemical groups have been directly associated with urease inhibition such as urea-based structural analogues as well as metal-binding groups. However, some of these may not actually provide the main contribution to the overall activity of inhibition but instead other less evident groups may do so. In this regard, we queried our data set with several active groups reported for urease inhibition (using SMARTS search with RDKit) and found groups generally associated with activity.

Due to their similarity to the tetrahedral configuration of the transition state of the enzymatic reaction carried by urease, organophosphorus compounds are competitive inhibitors that rank among the most potent molecules. However, in our data set, out of 183 such compounds, only 38.3% were active. Among them, phosphonic acid analogues, another well-known family, were also mostly inactive, with 79.1% being inactive out of 24 compounds. However, the subset of 31 phosphoramide compounds within the organophosphorus family was found to be often active, with 83.8% active molecules.

Urea- or thiourea-bearing compounds are another popular group of compounds owing to their similarity to urease’s native substrate. Among the 259 urea analogues in our data set, 55.5% were inactive. However, this included the 63 barbiturates, of which 69.8% are inactive, and 15 *N*-hydroxyureas, of which 33% were active molecules. Interestingly, the thiourea derivatives that are generally reported as being more potent than their urea counterparts were associated with relatively fewer actives (50.9%) out of 514 molecules (these included the 144 thiobarbiturates found to have 66.7% inactive compounds).

Even though the overall inactivity for all of the three families above is unexpected, this may be due to the common introduction of these groups into many different scaffolds. This is evident as these groups are found among the most potent inhibitors as well as in many inactive compounds. As a result, it is clear that the simple addition of these moieties does not drive activity.

On the other hand, the hydrazine substructure was found in 262 compounds, of which 77.4% were active molecules, and similarly, imines (97 molecules) were found to be enriched with active compounds (78.3%). Furthermore, even the hybrid thiosemicarbazides found in 261 compounds showed a striking 77.7% of active molecules. Another significant group that has been historically associated with urease inhibitors is sulfonamides, of which we identified 120 compounds with a considerable fraction of active molecules (78%). These classes of compounds are important urease inhibitors, but their use may not be straightforward as they are susceptible to hydrolysis and side effects. Another group that seemed to increase urease inhibition is the attachment of long carbon chains (superior to six carbons), which showed an enrichment of 65% of active molecules in a total of 123 molecules. However, as previously discussed, this may be *via* a noncompetitive mechanism of action.

Another commonly used group is the carboxylic acid found in 150 compounds but with no influence in generating actives (50% actives), and its ester form found in 232 compounds seems to be even more associated with inactive compounds (60.7% of inactive molecules).

Heterocyclic rings are also important for activity and are a part of many medicinal chemistry campaigns. Piperazine (N = 169) and pyridine (N = 224) are two of the ring-based groups most enriched with active compounds, with 53.8 and 58% active molecules, respectively. This is notable for pyridine, as this is a relatively under-reported group contributing to activity. Azoles, which also correspond to an important group in medicinal chemistry, were also associated with activity against ureases, having 59.8% actives from a total of 749 compounds. Upon applying clustering to these compounds (10 clusters generated in total), we observed a variability in activity from 15.7% of the actives for 51 compounds to 91.7% for 109 molecules, demonstrating the potential of this scaffold for urease inhibition, which nonetheless relies on fine-tuning (hence the variation). Even among the 5-membered heterocyclic rings, similar levels of actives were observed, with 59.5% of active compounds in a total of 923 compound. On the other hand, no significant enrichment was observed for annelated (1186 compounds) or bicyclic (1149 compounds) rings, with 50.3 and 50.7% of active molecules, respectively. Isatin analogues (66 compounds) did not greatly contribute to activity (49% active), whereas benzothiazoles (38 compounds) and thiophene (53) seem to be enriched with actives (89.8 and 71.6%, respectively).

Finally, any aryl-halogen (1277 compounds) was associated with activity between 51.6 and 60%, whereas aryl-thiols (77 compounds) were enriched with a remarkable 80.5% active molecules. On the contrary, the majority of 1,2-diphenols (147 compounds) were inactive (80.2% inactives).

### Translatability of Urease Inhibition between Species

The active site of ureases is well preserved, and it is often assumed that compounds tested against, for example, Jack bean urease will translate into activity against bacterial ureases. Some reports in the literature have tested the same compound against different ureases, and the data set we assembled contained 167 compounds tested against different species (at least two). Even though some did not have the same or similar IC_50_ or *K*_i_ values against different ureases, we observed that only a small portion of those compounds was found to be classified as active in a species and inactive in another species (20.1%). Overall, the compounds that showed different activities across species presented active groups related to binding directly to the nickel center (hydroxamic acids and diaminophosphinic acid) and a tendency to show a higher number of hydrogen-bonding groups (7.8 ± 1.9 *vs* 5.5 ± 1.4). Therefore, it is possible that the specificities of the H-bond networks dictate the right binding conformation to the metal of the pocket of different species.

### Comparison between the Chemical Space of Approved Drugs and Urease Inhibitors

**Figure 11:**
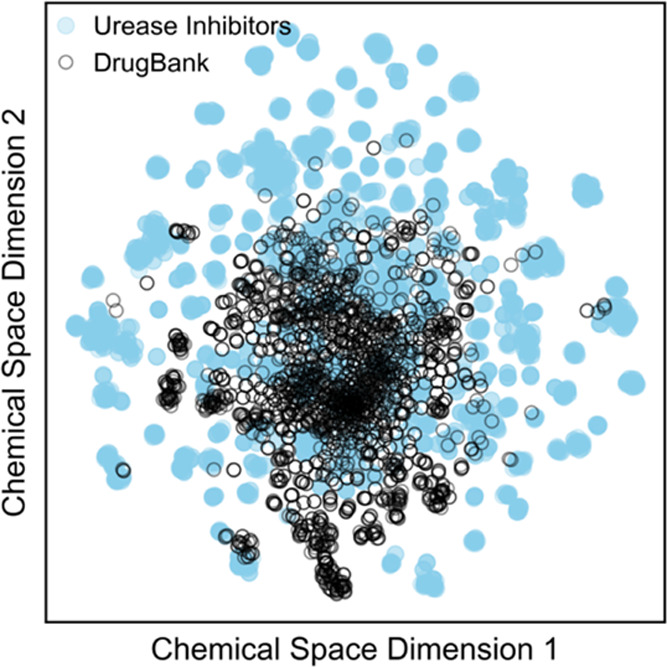
Chemical space of approved drugs (DrugBank) *versus* the urease inhibitor data set.

Another interesting feature to analyze is the distribution of the urease inhibitor data set with respect to approved drugs represented by DrugBank’s^49^ approved set (DrugBank version 5.1.8). This distribution is shown in Figure 11, revealing that the chemical space of approved drugs largely overlaps the known urease inhibitor space and, despite both having relatively the same size (2067 *vs* 3200 compounds), DrugBank shows a much lower diversity than urease inhibitors.

For example, antimicrobial drugs such as metronidazole and secnidazole (IC_50_ of 156 μM)^50^ have been reported to have an antiurease activity. With this in mind, we compared urease inhibitors with DrugBank to find high similarity matches. First, 19 urease compounds are also present in DrugBank (Table 2), which probably resulted from attempts of repurposing with the exception of acetohydroxamic acid (Lithostat), which is an approved urease inhibitor for urinary infections. Additionally, a total of 84 urease compounds show high similarity (T_c_ > 0.6) to at least one approved drug (Table S2).

## Conclusions

There has been an increasing interest in novel urease inhibitors, which has led to the testing of thousands of compounds against different ureases. Therefore, a large-scale in-depth analysis of developed work over the last few decades is paramount to help guide future drug discovery. Unfortunately, previous analyses of the activity of urease inhibitors were limited to a small number of compounds and, to our knowledge, no such large-scale survey exists. To bridge this gap, in the present work, we assembled the largest data set of urease inhibitors to date. This data set contained data on ureases from nine different species and 3200 unique compounds.

The urease inhibitors data set was used to characterize the chemical space of urease inhibitors and how it stands with respect to typical druggability filters. Compounds tested against ureases spanned a large range of molecular space, but the general physicochemical properties were found not to dictate activity. Temporal trends of compound development also revealed interesting observations that indicate, for example, some redundancy (accidental or purposeful) in the development of compounds within challenging scaffolds.

We also used machine learning tools such as clustering and decision trees to draw potential patterns that can be used to inform and guide medicinal chemists in the design and optimization of novel inhibitors. Particularly, we have disclosed scaffolds and chemical groups often associated with inhibitory activity as well as patterns that correlate with low activity. We also observed that simply adding chemical groups associated with activity generally does not result in improved activity but rather the scaffold seemed to drive this activity. On the other hand, under-reported groups such as pyridine were highlighted for their contribution to activity.

Furthermore, we extracted all activity cliffs present in the data set, which allows highlighting how much of the urease inhibitors’ chemical space is populated by unpredictable regions of structure–activity relationship. We have also reported examples of compounds that are desirable (safe bets) and undesirable (dead-ends) as starting points, since modifying them frequently leads to gain or loss of activity, respectively.

In conclusion, this work is a compilation of the current state of the art of urease inhibitors, where we provide insights into aspects and rules that can be used for a more rational initial stage of development of novel compounds. With this work, we aim to bias the research on this topic toward regions of chemical space more likely associated with activity.

## Supporting Information Available

Trends of the overall chemical properties of urease inhibitors in a historical perspective; distribution of different properties for actives, inactives, and the top 100 most active compounds; intracluster similarity distribution of the 50 clusters obtained from the full dataset; temporal trends of compound development within each cluster; normalized activity as a function of molecular weight for cluster 19; scaffolds from clustering; examples of dead-end and safe-bet compounds among urease inhibitors; urease inhibitors also present in the DrugBank (PDF)

## Notes

RDkit, Numpy, Scikit-learn, Pandas, Matplotlib, and Seaborn are freely available as Anaconda and/or pip packages (Python). Anaconda/Jupyter notebook is available for download and installation at https://docs.anaconda.com/anaconda/install. The different R packages used (iml, caret, mlr) are available for installation in CRAN. All raw data are available in the public domain (peer-reviewed publications, ChEMBL, and patents). Access to the full data set and analysis scripts is granted upon request.
