# Supplementary material for: Exploring the Chemical Space of Urease Inhibitors to Extract Meaningful Trends and Drivers of Activity
Source: J Chem Inf Model. 2022 Jun 6;62(15):3535–50. doi: 10.1021/acs.jcim.2c00150 (PMC9775197; doi:10.1021/acs.jcim.2c00150)
Supplement: Supplementary file 1 — ci2c00150_si_001.pdf [file ci2c00150_si_001.pdf]

SUPPORTING INFORMATION

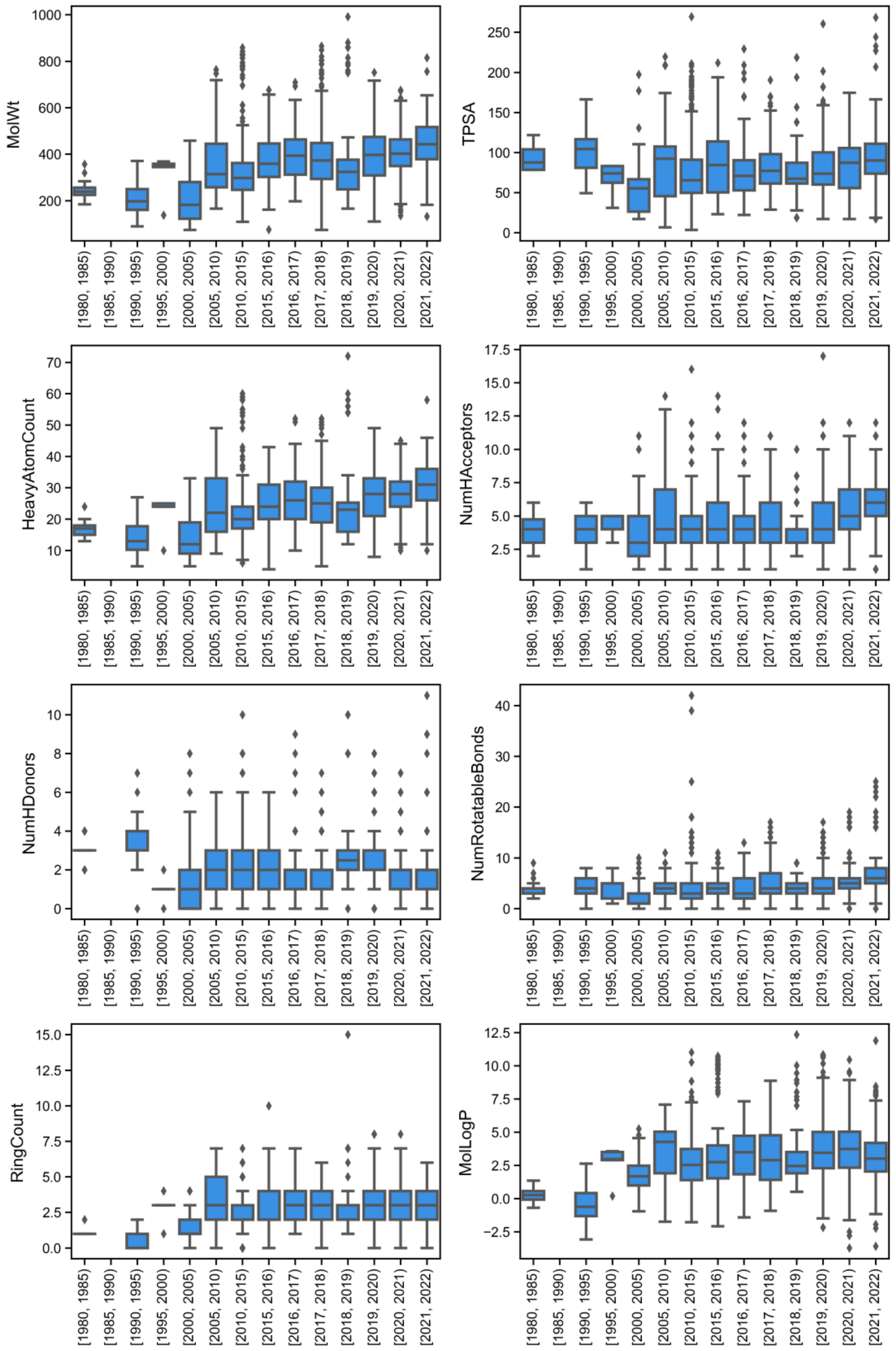

Figure S1. Trends of the overall chemical properties of urease inhibitors in a historical perspective.

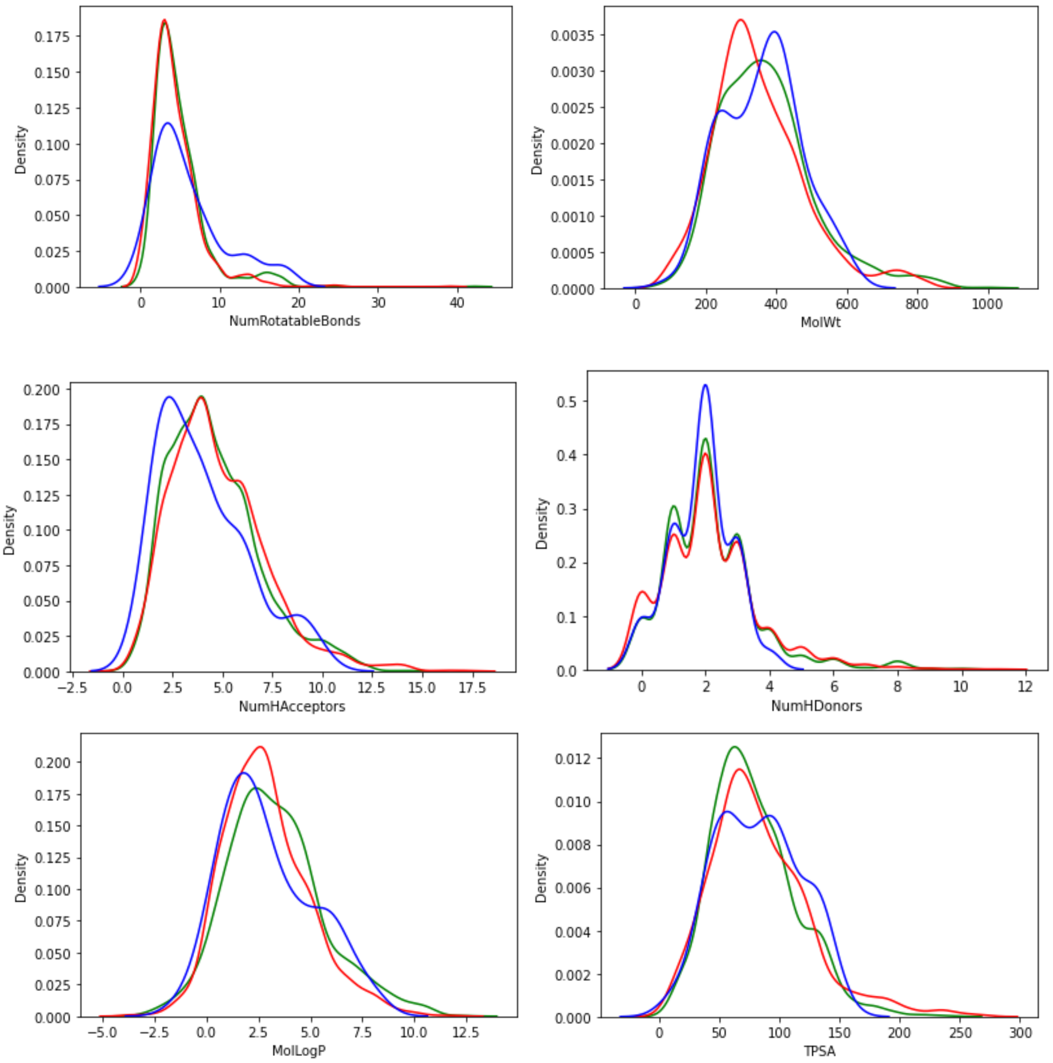

Figure S2. Distribution of different properties for active (green) and inactive (red) compounds and comparison to the top 100 most active compounds (blue).

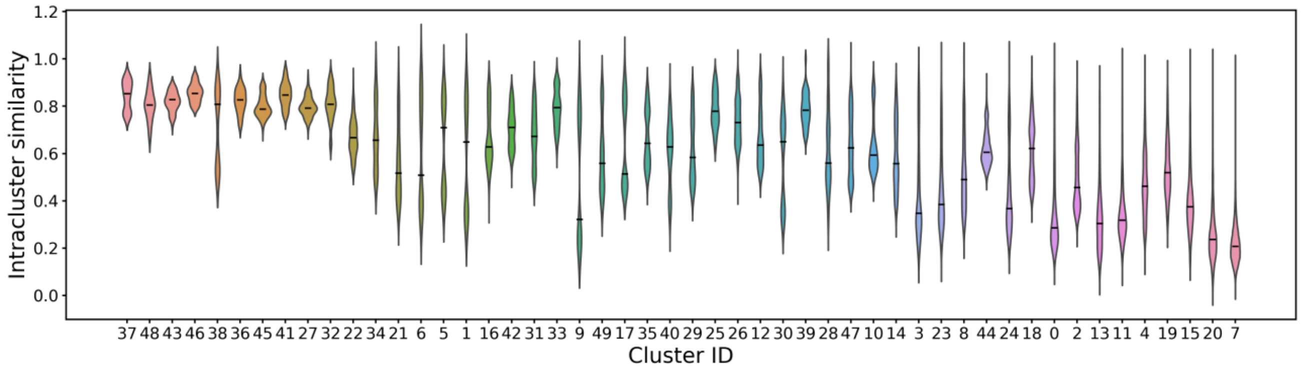

Figure S3. Intracluster similarity distribution of the 50 clusters obtained from the full dataset, with clusters sorted by ascending number of compounds per cluster. The black horizontal line across each distribution indicates the median intracluster similarity. The cluster with the lowest number of compounds (cluster 37) still had at least 10 compounds. The intracluster similarity distribution was obtained from calculating all-versus-all compound similarities in a given cluster.

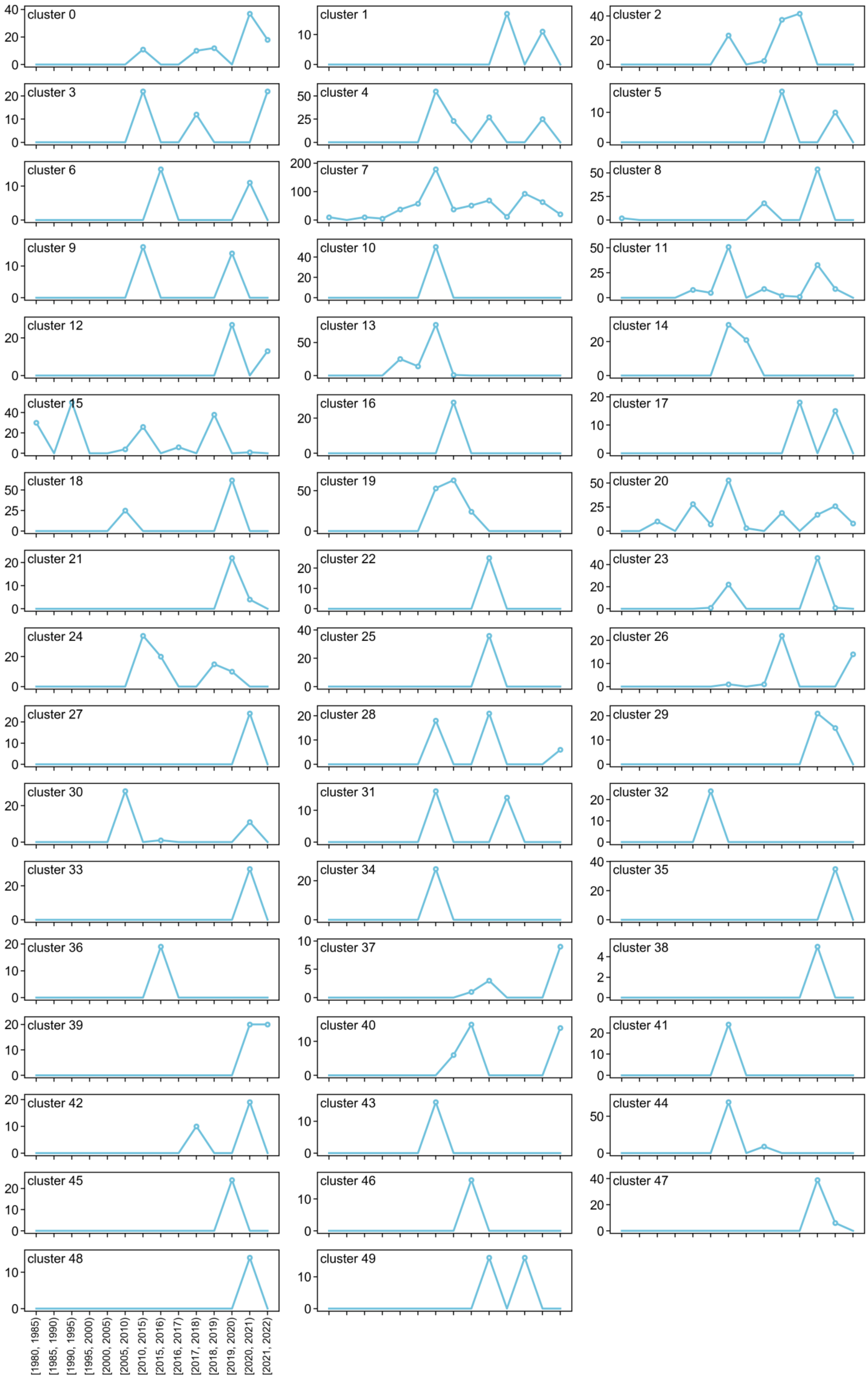

Figure S4. Temporal trends of compound development within each cluster where the Y-axis measures number of compounds and the X-axis measures time in years, and the X ticks correspond to the intervals shown in cluster 48's plot.

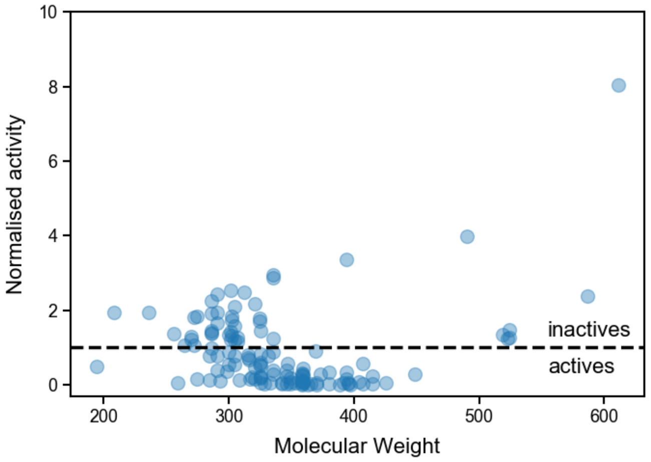

Figure S5. Normalised activity as a function of molecular weight for cluster 19. It is apparent that there is an interval of MW ideal for inhibitory activity. The Y axis was capped at a maximum of 10 to enable visualisation of the trend near the activity cut-off (normalised activity = 1). We hypothesize that too big or too small molecules do not properly fit into urease's catalytic pocket and thus do not effectively reduce the mobility of the flap helix.

Table S1. Scaffolds from clustering. MCS = Maximum common substructure (i.e. common scaffold); ICS min = minimum intracluster similarity; ICS med= median intracluster similarity.

Cluster ID	MCS pattern SMARTS	N comp.	% actives	ICS min.	ICS med.
0	c1ccccc1	88	95.5	0.11	0.28
1	O=[#6]-[#7]-c1ccccc1	28	42.9	0.26	0.64
2	[#6]\[#6]=[#6]/[#6]-[#6]-1[N,C][#6](=[S,O])-[#7]-[#6]=[#6]-1-[#6]=O	106	55.7	0.25	0.45
3	[#6]-c1ccccc1	56	48.2	0.14	0.34
4	[#6]=[#6]-1-[#6](=O)-[#7]-[#6](=[O,S])-[#7]-[#6]-1=O	130	34.6	0.13	0.46
5	[#6]=[#6]-c1cc2ccccc2oc1=O	27	85.2	0.34	0.70
6	[#6]-1-[#6]-[#6](-[#6]-2=[#7]-[#6](-[#6](-[#7]-2)-c2ccccc2)-c2ccccc2)=[#6](-[#7]-1)-c1ccccc1	26	65.4	0.27	0.50
7	--	674	49.4	0.0	0.20
8	O=[#6](-[#7]-[#7]-[#6]=[O,S])-[#6]-1=[#6]-[#6]=[N,C][#6]=[#6]-1	74	67.6	0.22	0.48
9	[#6]-[#7]-1-[#6]=[#7]-[#6](=O)\[#6](=[#6]\[#7])-[#6]-1=O	30	50.0	0.19	0.32
10	[#6]-[#7](-[#8])-[#6](=O)-[#6]C1([#8])[#6]=[#6](-[#8]-c2ccccc12)-c1ccccc1	50	20.0	0.45	0.59
11	[#6]-c1ccccc1	118	21.2	0.08	0.31
12	[#6]-n1c(-[#6]-c2ccccc2)nc2ccccc2c1=O	40	100	0.48	0.63
13	[N,S]1[#6]=[N,S][N,S]=[#6]1	117	27.4	0.05	0.30
14	[#6]-c1nccn1-[#6]-[#6]-[#8]	51	15.7	0.31	0.55
15	[#8]-[#7]-[#6]=O	155	71.0	0.10	0.37
16	[#6]C1([#6])[#6]-[#6](-[#8])=[#6](-[#6](-[#6]-[#6]=O)-c2ccccc2)-[#6](=O)-[#6]1	29	0.0	0.38	0.62
17	[#6]-[#6]-1=[#6]-[#6]=[#6](-[#6]=[#6]-1)-[#6](-[#6]-1=[#6]-[#7]-[#6]-2=[#6]-[#6]=[#6]-[#6]=[#6]-1-2)-[#6]-1=[#6]-[#7]-[#6]-2=[#6]-1-[#6]=[#6]-[#6]=[#6]-2	33	57.6	0.42	0.51
18	[#8]-c1c(-[#6]-c2c(-[#8])c3ccccc3oc2=O)c(=O)oc2ccccc12	87	19.5	0.35	0.62
19	c1ccccc1; O=[#6]-[#7]-[#6](=S)-[#7]-c1ccccc1	140	56.4	0.24	0.51
20	--	175	34.9	0.0	0.23
21	[#7]=c1nc(cs1)-c1ccccc1	26	73.1	0.31	0.51
22	[#6]-c1ccc2nc(nc2c1)-c1ccccc1	25	20	0.51	0.66
23	[#8]-c1ccccc1-[#8]	70	11.4	0.12	0.38
24	[#7]-[#6]-1=[#6]-[#6]=[#6]-[#6]=[#6]-1	79	68.4	0.16	0.36

25	[#6]-[#6](-[#7])-[#6](=O)-[#7]-1-[#6]-[#6]-[#6]-[#6]-1-[#6](=O)-[#7]-1-[#6]-[#6]-[#7](-[#6]-[#6]-1)-c1ccccc1	36	47.2	0.61	0.77
26	[Cl]-c1cc2c(cc1-[#7]-1-[#6]-[#6]-[#7]-[#6]-[#6]-1)ncc(-[#6]=O)c2=O	38	59.9	0.43	0.73
27	O=[#6](-[#6]-[#8]-c1cc(=O)oc2ccccc12)-[#7]\[#7]=[#6]\c1ccccc1	24	33.3	0.68	0.79
28	[#6]-[#6]-[#6]-[#6]-[#6]-[#6]-[#6]-[#6]-[#6]-[#6]-[#6]-[#6]=O	45	86.7	0.27	0.55
29	[#6]\[#6](=[#7]/[#7]-[#6](-[#7])=S)-c1ccccc1	36	88.9	0.39	0.58
30	[#7]-c1nc(cs1)-c1ccccc1	40	82.5	0.27	0.64
31	[#7]-[#6](=S)-[#7]\[#7]=[#6]-1/[#6](=O)-[#7]-c2ccccc-12	30	56.7	0.45	0.67
32	[#7]-[#6](-[#6]-[#6]-[#6]=O)-[#6](=O)-[#7]-1-[#6]-[#6]-[#7](-[#6]-[#6]-1)-c1nsc2ccccc12	24	54.2	0.62	0.80
33	[#8]-c1ccc2c(oc(\[#6]=[#6]\[#6]=[#6])cc2=O)c1\[#6]=[#7]/[#7]-[#6]=O	30	90.0	0.58	0.79
34	[#8]-[#6]-c1nnc(-[#7]-c2ccccc2)o1	26	38.5	0.43	0.65
35	[#6]\[#6]=[#6](/[#6]=[#6])\[#6]=[#7]/c1ccc(cc1)S(=O)(=O)[#7]-[#6]=[#7]	35	77.1	0.45	0.64
36	Clc1ccc2-[#7]-[#6](=O)\[#6](=[#6]\c3ccc(cc3)-[#6](=O)-[#7]\[#7]=[#6]\c3ccccc3)-c2c1	19	10.5	0.70	0.82
37	[#6]-[#6]-1-[#6]-[#8]-c2c(-[#7]-3-[#6]-[#6]-[#7](-[#6])-[#6]-[#6]-3)c(F)cc3-[#6]-[#6](-[#6]=O)=[#6]-[#7]-1-c23	13	92.3	0.75	0.85
38	[#6]C1([#6])[#6]-[#6]-2=[#6](-[#6](-[#6]3=[#6](-[#6]C([#6])([#6])[#6]\[#6]-3=[#7]/[#7])-[#7]-2)-c2ccccc2)\[#6](-[#6]1)=[#7]\[#7]	17	5.9	0.49	0.80
39	[#6]-[#6]-[#6]-[#6]-[#6]-[#6]-[#6]-[#6]-[#6]-[#6]-[#6]-[#6]-[#6]-[#6]-[#6]-c1nnc(-[#16])n1-[#7]=[#6]	40	77.5	0.63	0.78
40	[#6]-[#6]-[#7]-1-[#6]-[#7](-[#6]-[#6])-[#6](=O)-[#6](-[#6](-[#6])-[#6]-2-[#6](=O)-[#7](-[#6])-[#6]-[#7](-[#6])-[#6]-2=O)-[#6]-1=O	35	34.3	0.26	0.62
41	[#6]-[#6]-[#6@H](-[#7]-[#6](=O)-[#6]-[#7]-[#6]=S)-[#6](=O)-[#7]-[#6]-[#6](=O)-[#7]-[#6@@H](-[#6](-[#6])-[#6])-[#6](=O)-[#7]-1-[#6]-[#6]-[#6]-[#6@H]-1-[#6](=O)-[#7]-1-[#6]-[#6]-[#7](-[#6]-[#6]-1)-c1nsc2ccccc12	24	33.3activity value	0.73	0.84
42	[#6]-[#6](-[#6])-[#6]-c1ccc(cc1)-[#6](-[#6])-[#6](-[#7])=O	29	79.3	0.50	0.71
43	[#6]-n1c2ccccc2c2cc(ccc12)-c1cc(\[#6]=[#6]\[#6]=[#6])nc(-[#7])n1	16	6.2	0.71	0.82
44	[#6]-[#7](-[#8])-[#6](=O)-[#6]-[#6](-[#8])-c1ccccc1	78	19.2	0.47	0.60
45	[#6]-[#6](-[#6])-[#6](-[#7])-[#6]-[#6](-[#8])-[#6]-[#8]-c1ccc(-[#6]-[#6](-[#7])=O)cc1	24	29.2	0.68	0.78
46	[#7]-[#6](=S)-[#7]-[#7]=[#6]-[#6]-1=[#6]-[#6]=[#6]-[#6]=[#6]-1-[#8]-[#6]-[#6]-1=[#6]-[#6]-2=[#6]-[#6]=[#6]-[#6]=[#6]-2-[#8]-[#6]-1=O	16	43.8	0.78	0.85
47	[#6]-[#6]-c1ccc2ccc(-[#8]-[#6])cc2c1	45	24.4	0.42	0.62
48	[#8-]-[#7+](=O)-c1cc(Cl)c2cc(oc2c1)-[#6](=O)-[#7]-1-[#6]-[#6]-[#16]-[#6]-1=O	14	92.9	0.65	0.80
49	[#6]-n1cnc2cc(Cl)c(Cl)cc12	32	84.4	0.34	0.55

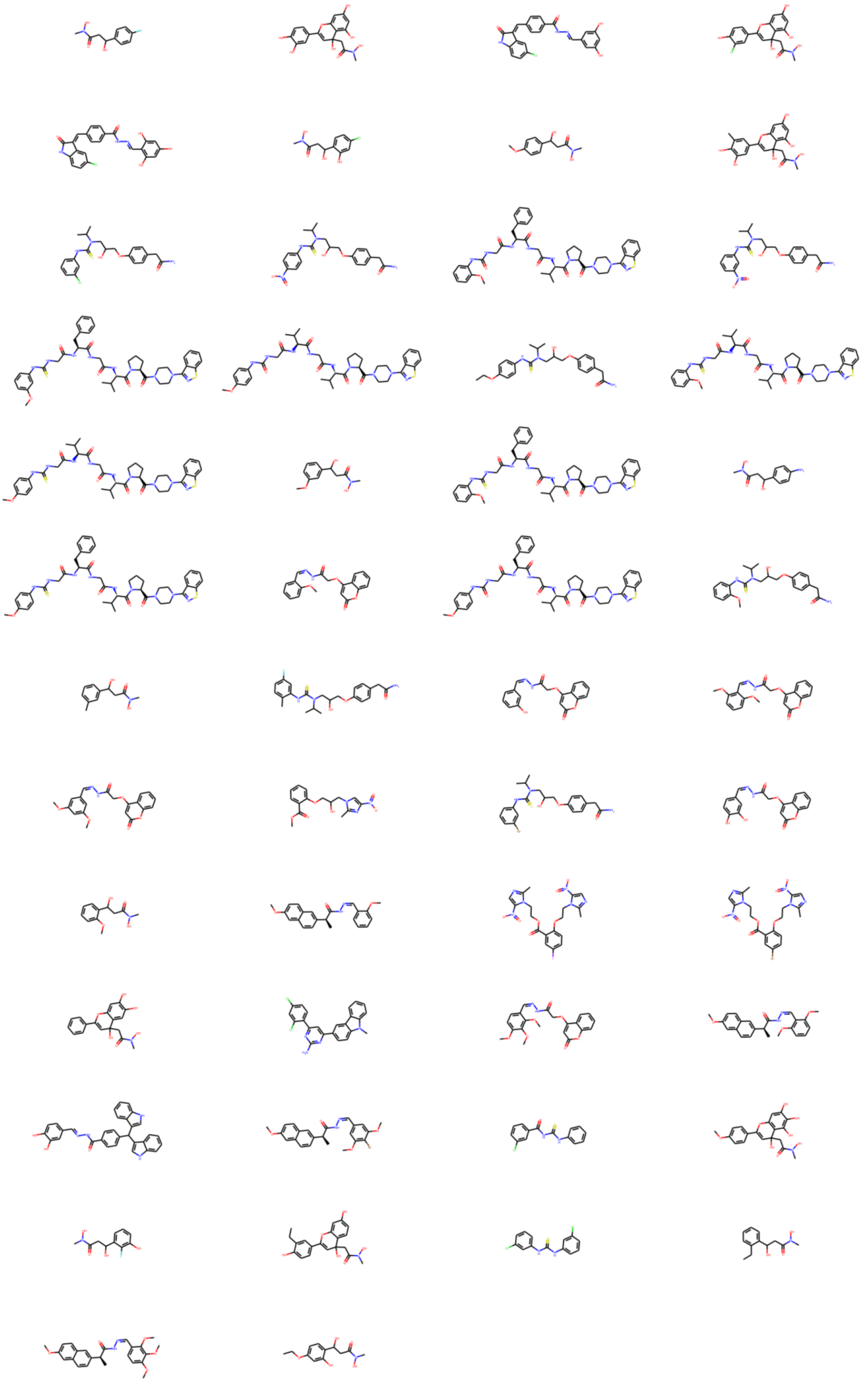

Figure S6. Example of dead-end molecules (part 1, cont.).

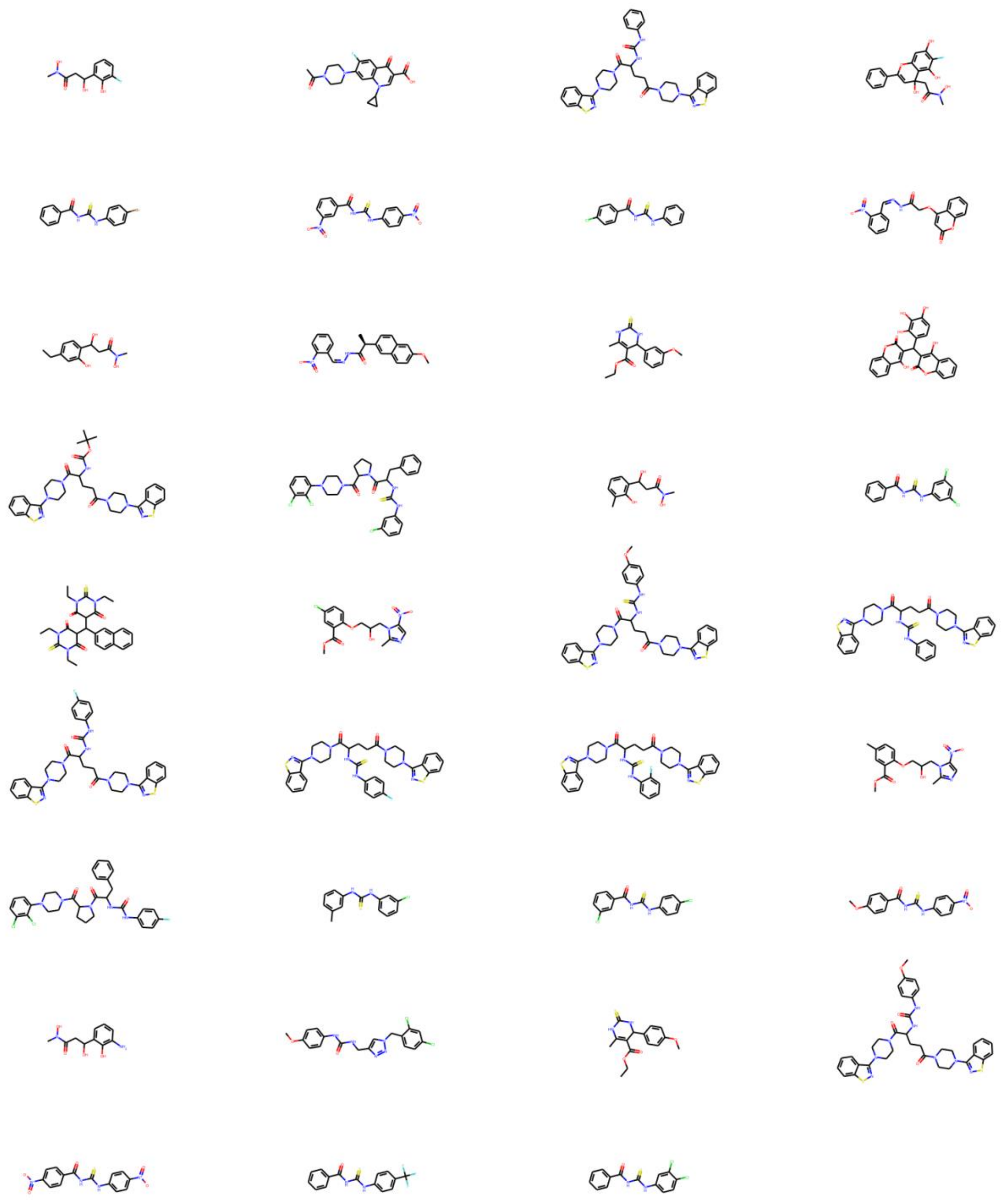

Figure S6. Example of dead-end molecules (part 2).

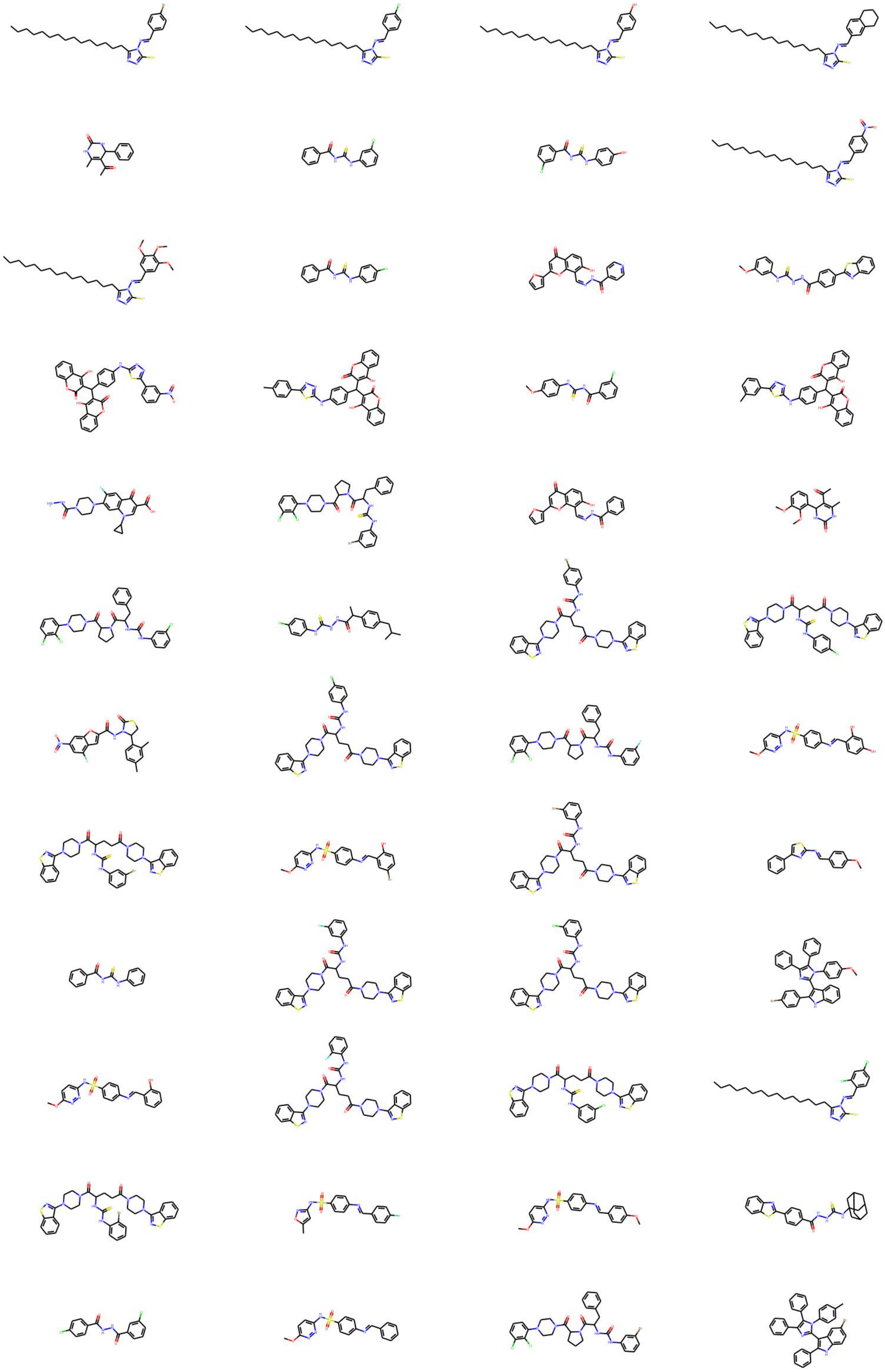

Fig S7. Example of safe bets (part 1, cont.).

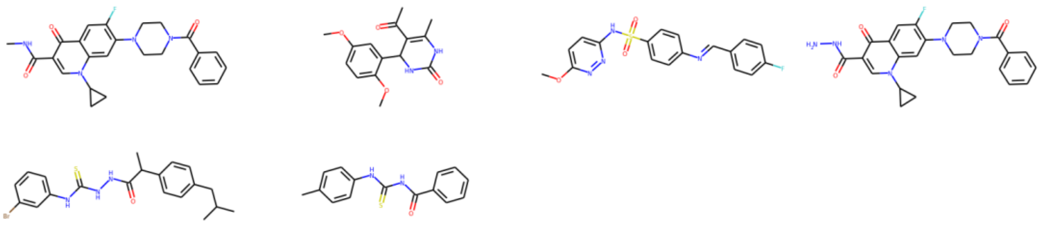

Fig S7. Example of safe bets (part 2, cont.).

Table S2. Urease inhibitors also present in the DrugBank.

Name	SMILES
**Deferiprone**	O=C\1C(\O)=C(/N(/C=C/1)C)C
**Phenylalanine**	[NH3+][C@@H](CC1=CC=CC=C1)C([O-])=O
**Ropinirole**	O=C2Nc1cccc(c1C2)CCN(CCC)CCC
**Pyroglutamic acid**	O=C(O)[C@H]1NC(=O)CC1
**Acetohydroxamic acid**	O=C(NO)C
**Hydroxycarbamide**	O=C(N)NO
**Etacrynic acid**	O=C(O)COc1c(Cl)c(Cl)c(cc1)C(=O)C(=C)CC
**Mannitol**	O[C@H]([C@H](O)CO)[C@H](O)[C@H](O)CO
**Atenolol**	O=C(N)Cc1ccc(cc1)OCC(O)CNC(C)C
**Ofloxacin**	Fc4cc1c2N(/C=C(\C1=O)C(=O)O)C(COc2c4N3CCN(C)CC3)C
**Captopril**	O=C(O)[C@H]1N(C(=O)[C@H](C)CS)CCC1
**Rutin**	CC1C(C(C(C(O1)OCC2C(C(C(C(O2)OC3=C(OC4=CC(=CC(=C4C3=O)O)O)C5=CC(=C(C=C5)O)O)O)O)O)O)O)O
**Dicoumarol**	O=C1Oc2ccccc2C(O)=C1CC3=C(O)c4ccccc4OC3=O
**Isoniazid**	C1=CN=CC=C1C(=O)NN
**Secnidazole**	[O-][N+](=O)c1cnc(n1CC(O)C)C
**Berberine**	O1c2c(OC1)cc5c(c2)c4cc3ccc(OC)c(OC)c3c[n+]4CC5
**Cianidanol**	O[C@H]1CC2=C(O)C=C(O)C=C2O[C@@H]1C1=CC=C(O)C(O)=C1
